# Supplementary material for: Phosphorylation of the F-BAR protein Hof1 drives septin ring splitting in budding yeast
Source: Nat Commun. 2024 Apr 22;15:3383. doi: 10.1038/s41467-024-47709-3 (PMC11035697; doi:10.1038/s41467-024-47709-3)
Supplement: Supplementary file 1 — Supplementary Information [file 41467_2024_47709_MOESM1_ESM.pdf]

Phosphorylation of the F-BAR protein Hof1 drives septin ring splitting in budding yeast

Varela Salgado et al.

SUPPLEMENTARY MATERIAL

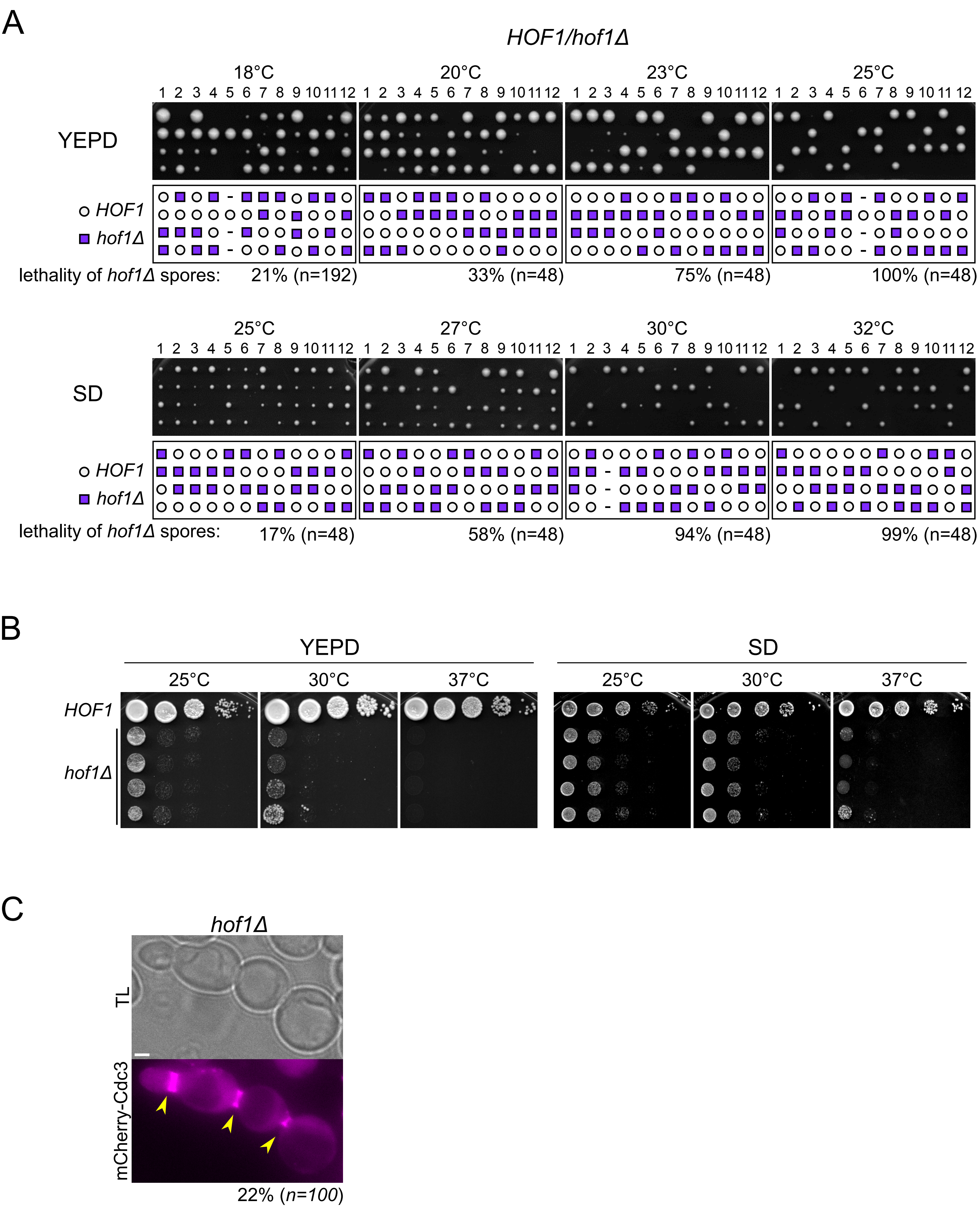
A
HOF1/hof1Δ
18°C
20°C
23°C
25°C
1 2 3 4 5 6 7 8 9 10 11 12
YEPD
○ HOF1
■ hof1Δ
lethality of hof1Δ spores:
21% (n=192)
33% (n=48)
75% (n=48)
100% (n=48)
25°C
27°C
30°C
32°C
SD
17% (n=48)
58% (n=48)
94% (n=48)
99% (n=48)
B
YEPD
SD
25°C
30°C
37°C
HOF1
hof1Δ
C
hof1Δ
TL
mCherry-Cdc3
22% (n=100)

Figure S1. Growth conditions influence the viability of *hof1Δ* cells.

A: Tetrad analysis of *HOF1/hof1Δ* heterozygous diploid cells after sporulation and tetrad dissection on YEPD or SD medium and incubation at different temperatures. Numbers indicate different tetrads. **B:** Serial dilutions of *HOF1* or *hof1Δ* cells were spotted on YEPD and SD medium and incubated at the indicated temperatures. **C:** *hof1Δ* cells expressing mCherry-Cdc3 were grown in YEPD at 30°C and imaged. TL: transmitted light. Scale bar: 2 µm.

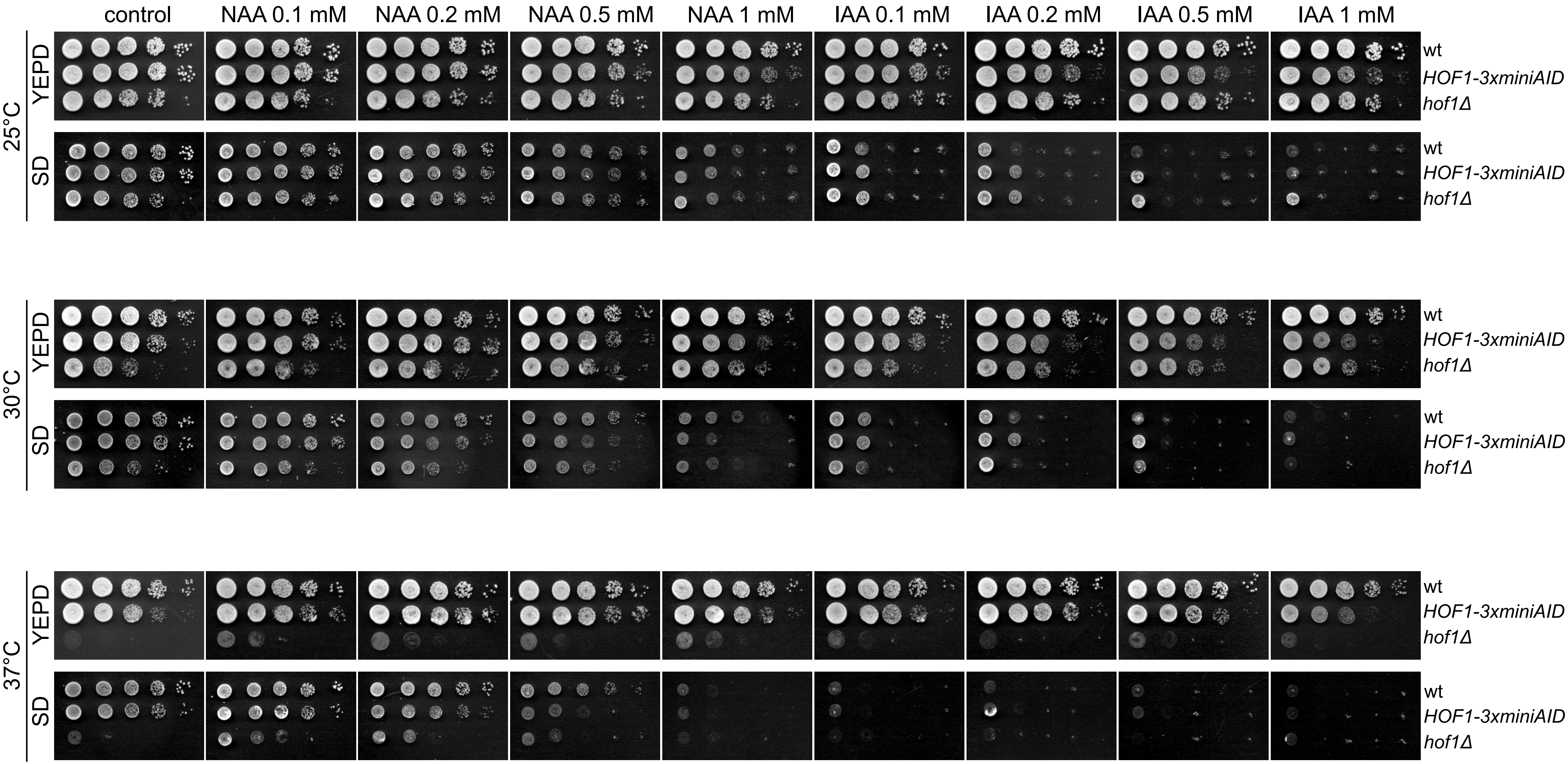
control
NAA 0.1 mM
NAA 0.2 mM
NAA 0.5 mM
NAA 1 mM
IAA 0.1 mM
IAA 0.2 mM
IAA 0.5 mM
IAA 1 mM
25°C
30°C
37°C
YEPD
SD
wt
HOF1-3xminiAID
hof1Δ

Figure S2. Viability of *HOF1-3XminiAID* cells under different conditions. Serial dilutions of wild type, *HOF1-3XminiAID* and *hof1Δ* cells were spotted on YEPD or SD plates containing the indicated concentrations of NAA (1-naphtaleneacetic acid) or IAA (3-indolacetic acid) and incubated at different temperatures. We note that in general IAA is more toxic than NAA for wild type cells.

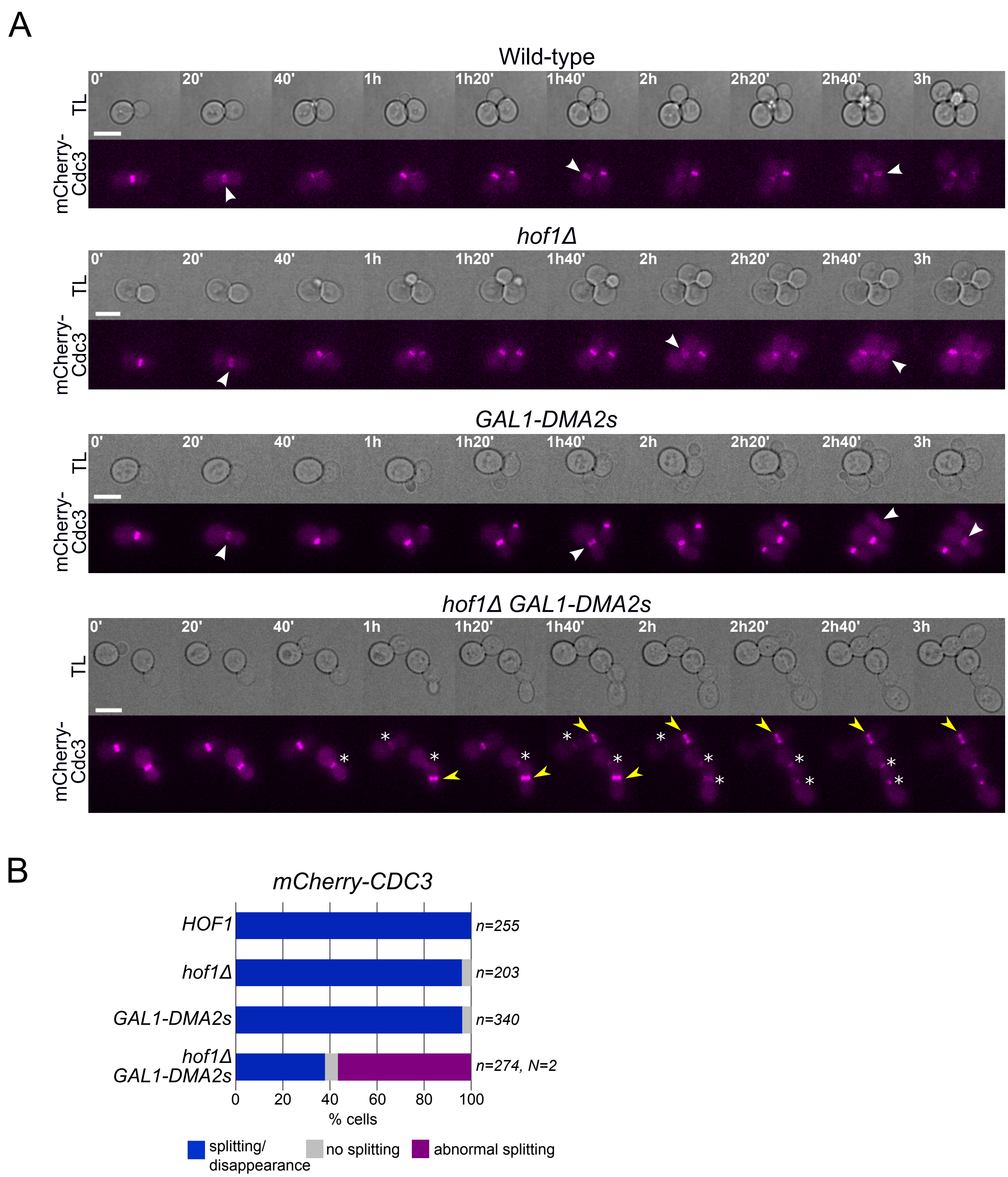
A
Wild-type
0'
20'
40'
1h
1h20'
1h40'
2h
2h20'
2h40'
3h
TL
mCherry-Cdc3
hof1Δ
GAL1-DMA2s
hof1Δ GAL1-DMA2s
B
mCherry-CDC3
HOF1
hof1Δ
GAL1-DMA2s
hof1Δ GAL1-DMA2s
n=255
n=203
n=340
n=274, N=2
0
20
40
60
80
100
% cells
splitting/ disappearance
no splitting
abnormal splitting

Figure S3. *HOF1* deletion causes a synthetic septin splitting defect in combination with moderate *DMA2* overexpression.

A-B: Cells with the indicated genotypes and expressing mCherry-Cdc3 were grown in SD-raffinose and induced for 90 min with galactose before imaging in SD-raffinose/galactose at 30°C. Septin splitting and splitting defects have been quantified in cells undergoing mitotic exit and a new round of budding. White arrowheads indicate normal septin ring splitting/clearance at cytokinesis; yellow arrowheads indicate septin ring formation in the absence of previous septin ring splitting; asterisks indicate partial septin clearance.

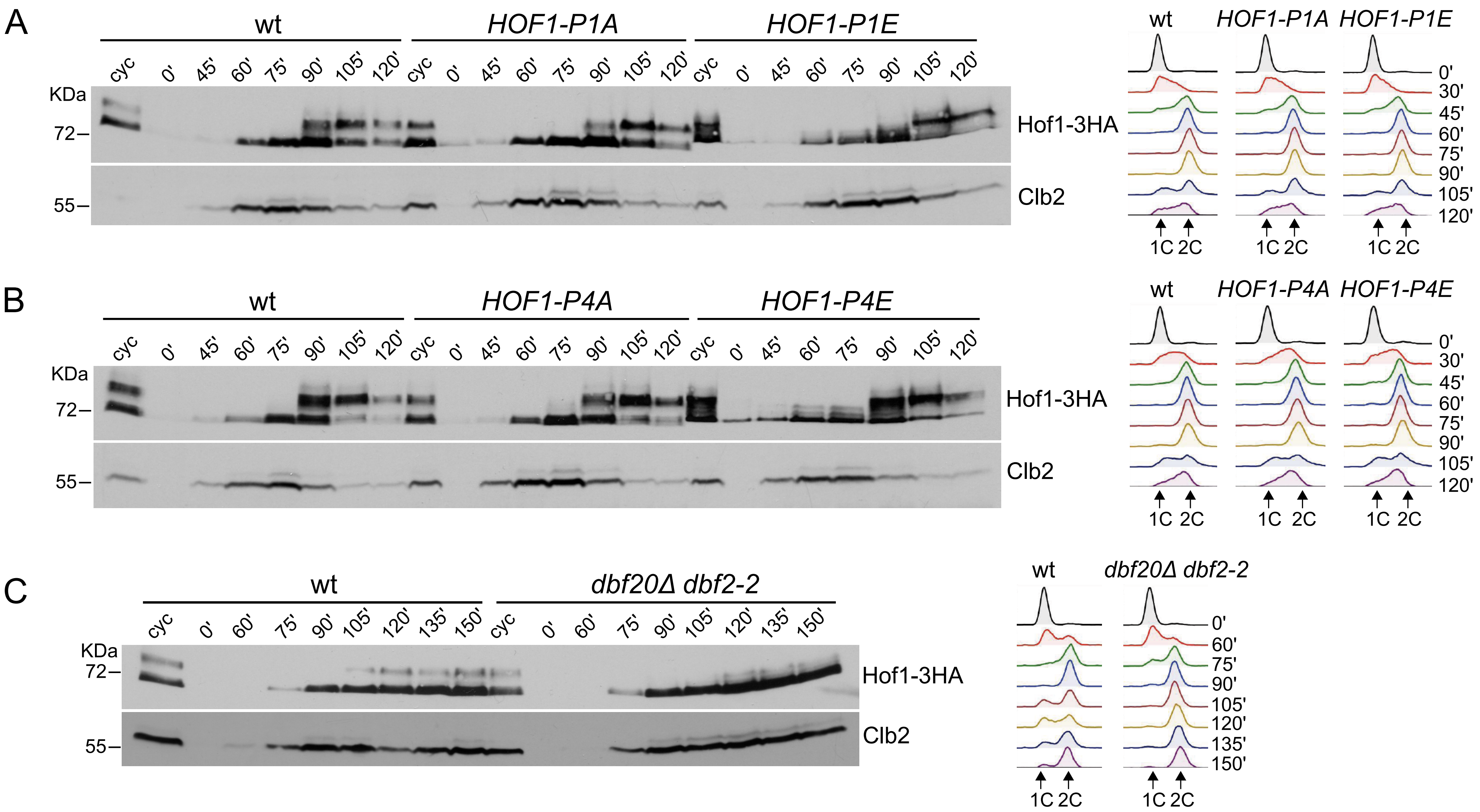
A
wt
HOF1-P1A
HOF1-P1E
cyc 0' 45' 60' 75' 90' 105' 120'
KDa
72–
55–
Hof1-3HA
Clb2
0'
30'
45'
60'
75'
90'
105'
120'
1C 2C
B
wt
HOF1-P4A
HOF1-P4E
Hof1-3HA
Clb2
C
wt
dbf20Δ dbf2-2
cyc 0' 60' 75' 90' 105' 120' 135' 150'
Hof1-3HA
Clb2
150'
1C 2C

Figure S4. Hof1 phosphorylation during the cell cycle in different mutants. A-B: Cells bearing wild type (wt) *HOF1* or the indicated *HOF1* mutant alleles tagged with 3HA epitopes were arrested in G1 by alpha factor and then released in fresh YEPD at 25°C (time=0). Cell samples were collected at different time points for western blot analysis of Hof1-3HA protein levels (left) and for analysis of DNA contents by flow cytometry (right). The mitotic cyclin Clb2 was used as a marker of mitotic entry/progression. Cyc: cycling cells. **C:** Wild type (wt) and *dbf20Δ dbf2-2* mutant cells expressing Hof1-3HA were grown to logarithmic phase at 25°C, arrested in G1 by alpha factor and then released in fresh medium at 37°C. Cell samples at different time points were processed as in A-B.

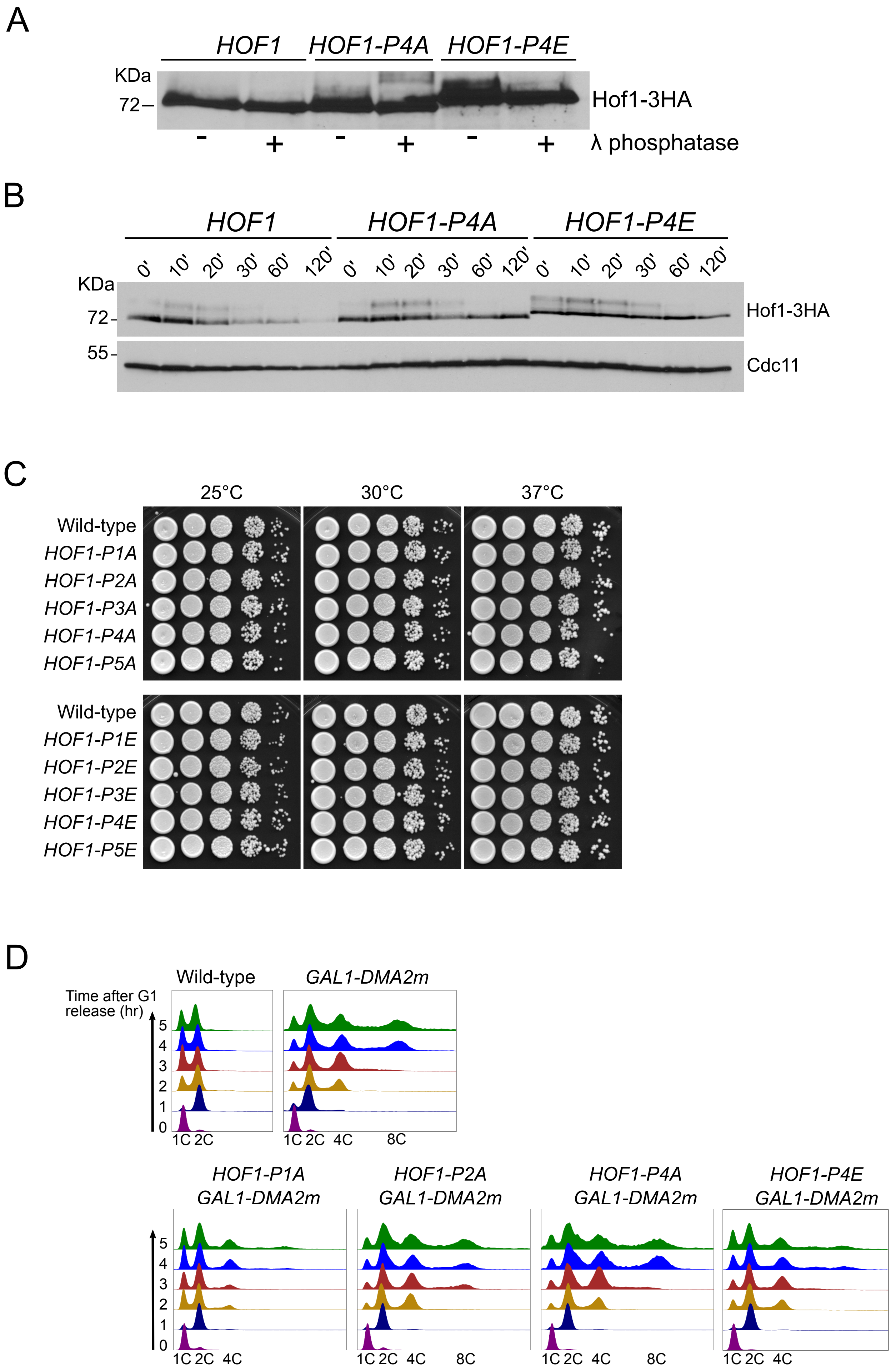
A
HOF1
HOF1-P4A
HOF1-P4E
KDa
72
Hof1-3HA
-
+
-
+
-
+
λ phosphatase
B
HOF1
HOF1-P4A
HOF1-P4E
0' 10' 20' 30' 60' 120'
0' 10' 20' 30' 60' 120'
0' 10' 20' 30' 60' 120'
KDa
72
55
Hof1-3HA
Cdc11
C
25°C
30°C
37°C
Wild-type
HOF1-P1A
HOF1-P2A
HOF1-P3A
HOF1-P4A
HOF1-P5A
Wild-type
HOF1-P1E
HOF1-P2E
HOF1-P3E
HOF1-P4E
HOF1-P5E
D
Wild-type
GAL1-DMA2m
Time after G1 release (hr)
5
4
3
2
1
0
1C 2C
1C 2C 4C 8C
HOF1-P1A GAL1-DMA2m
HOF1-P2A GAL1-DMA2m
HOF1-P4A GAL1-DMA2m
HOF1-P4E GAL1-DMA2m
5
4
3
2
1
0
1C 2C 4C
1C 2C 4C 8C
1C 2C 4C 8C
1C 2C 4C

Figure S5. Effects of *HOF1* phosphorylation mutants on Hof1 protein stability and cytokinesis.

A: Hof1-3HA was immunoprecipitated from cells extracts of wild type (*HOF1*) or *HOF1-P4A* and *HOF1-P4E* mutants. Immunoprecipitates were treated with lambda phosphatase or with buffer alone and run on SDS page for western blot analysis of Hof1-3HA. Note that the electrophoretic mobility shift of the Hof1-P4E-3HA protein remains unaffected upon dephosphorylation. **B:** wild type (*HOF1*) and *HOF1-P4A* and *HOF1-P4E* cell cultures were treated with cycloheximide at time=0. At the indicated times cell samples were collected for western blot analysis of Hof1-3HA. Cdc11 was used as loading control. **C:** Serial dilutions of cells with the indicated genotypes were spotted on YEPD and incubated at the indicated temperatures. **D:** *GAL1-DMA2m* cells carrying the indicated *HOF1* alleles were grown in YEPR, arrested in G1 by α-factor and induced with 1% galactose 30 minutes before release in YEPRG in parallel to cells shown in Fig. 2G. At the indicated times cells were withdrawn for flow-cytometric analysis of DNA contents. Histograms for wild type and *GAL1-DMA2m* controls are duplicated from Fig. 2G.

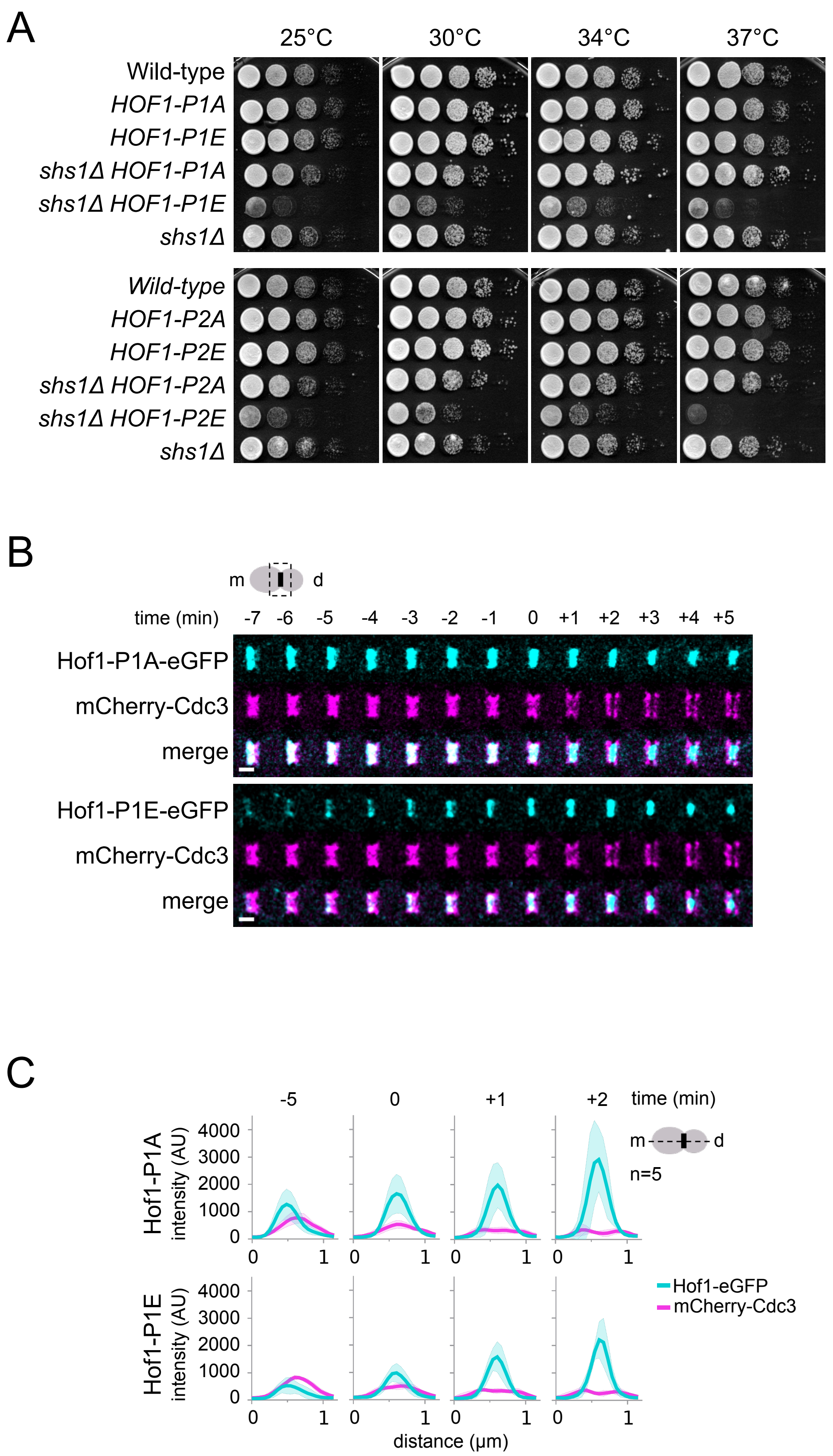
A
25°C
30°C
34°C
37°C
Wild-type
HOF1-P1A
HOF1-P1E
shs1Δ HOF1-P1A
shs1Δ HOF1-P1E
shs1Δ
Wild-type
HOF1-P2A
HOF1-P2E
shs1Δ HOF1-P2A
shs1Δ HOF1-P2E
shs1Δ
B
m
d
time (min) -7 -6 -5 -4 -3 -2 -1 0 +1 +2 +3 +4 +5
Hof1-P1A-eGFP
mCherry-Cdc3
merge
Hof1-P1E-eGFP
mCherry-Cdc3
merge
C
-5
0
+1
+2
time (min)
m
d
n=5
Hof1-P1A
intensity (AU)
Hof1-P1E
intensity (AU)
4000
3000
2000
1000
0
0
1
distance (µm)
Hof1-eGFP
mCherry-Cdc3

Figure S6. Phospho-mimicking *HOF1* alleles impair Hof1 binding to septins.

A: Serial dilutions of cells with the indicated genotypes were spotted on YEPD and incubated at the indicated temperatures. **B-C:** Cells expressing mCherry-Cdc3 and the indicated Hof1-eGFP proteins were filmed in SD medium at 30°C. Images were deconvolved with the Andor Fusion software to resolve single and double Hof1 rings. Graphs in C) represent Hof1-eGFP and mCherry-Cdc3 average fluorescence intensities (n=5) along a line spanning the bud neck. Time 0 is the time of septin ring splitting. Shaded curves: s.d.

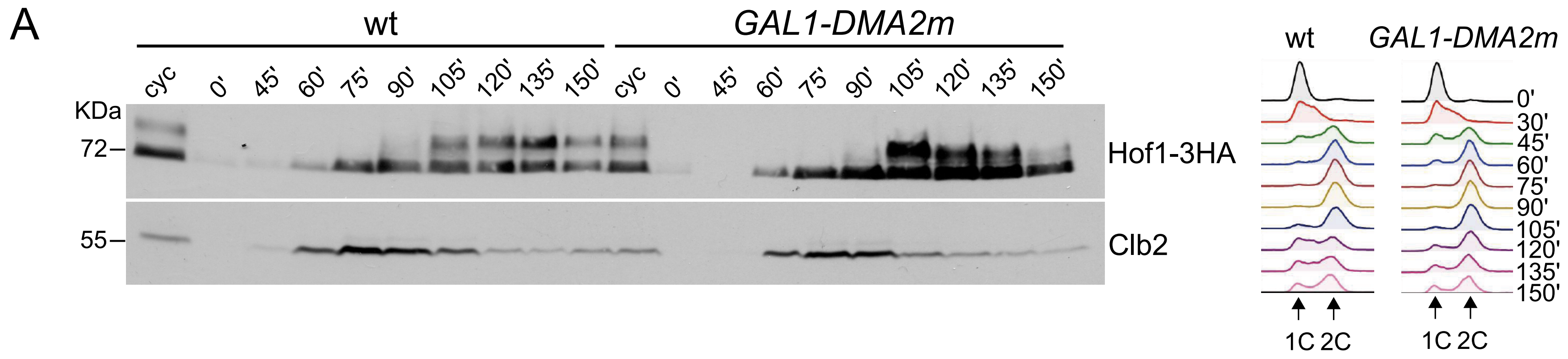
A
wt
GAL1-DMA2m
cyc 0' 45' 60' 75' 90' 105' 120' 135' 150' cyc 0' 45' 60' 75' 90' 105' 120' 135' 150'
KDa
72
55
Hof1-3HA
Clb2
wt
GAL1-DMA2m
0'
30'
45'
60'
75'
90'
105'
120'
135'
150'
1C 2C
1C 2C

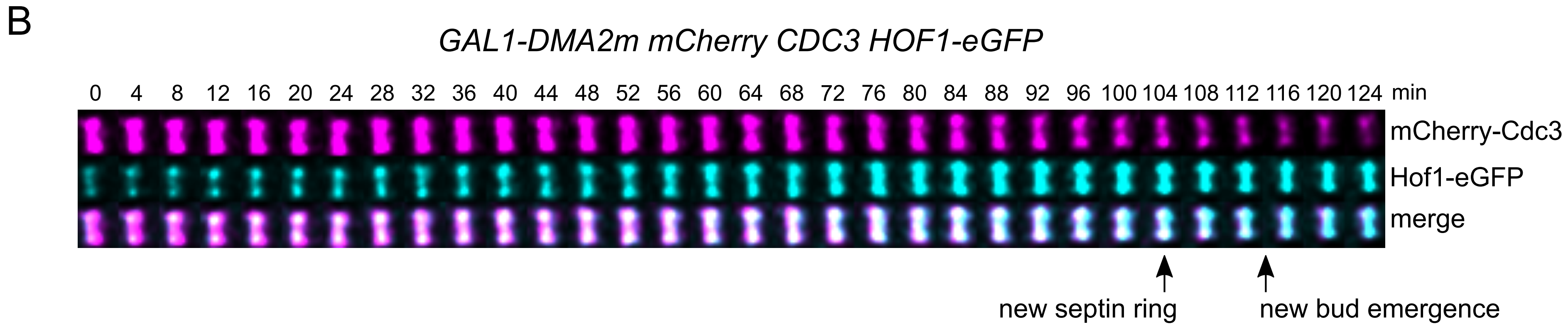
B
GAL1-DMA2m mCherry CDC3 HOF1-eGFP
0 4 8 12 16 20 24 28 32 36 40 44 48 52 56 60 64 68 72 76 80 84 88 92 96 100 104 108 112 116 120 124 min
mCherry-Cdc3
Hof1-eGFP
merge
new septin ring
new bud emergence

Figure S7. ***DMA2*** **overexpression leads to increased Hof1 protein levels in mitosis. A:** Wild type and *GAL1-DMA2m* cells expressing Hof1-3HA were grown in YEPR at 25°C, arrested in G1 by alpha factor and induced by 1% galactose 30 minutes before their release in YEPRG (time=0). Cell samples were collected at the indicated time points for western blot analysis of Hof1-3HA protein levels (left) and for analysis of DNA contents by flow cytometry (right). The mitotic cyclin Clb2 was used as a marker of mitotic entry/progression. Cyc: cycling cells. **B:** *GAL1-DMA2m* cells expressing mCherry-Cdc3 and Hof1-eGFP were grown in SD-raffinose and induced for 60 min with galactose before imaging in SD-raffinose/galactose at 30°C. Max-projected images were deconvolved and cropped around the bud neck.

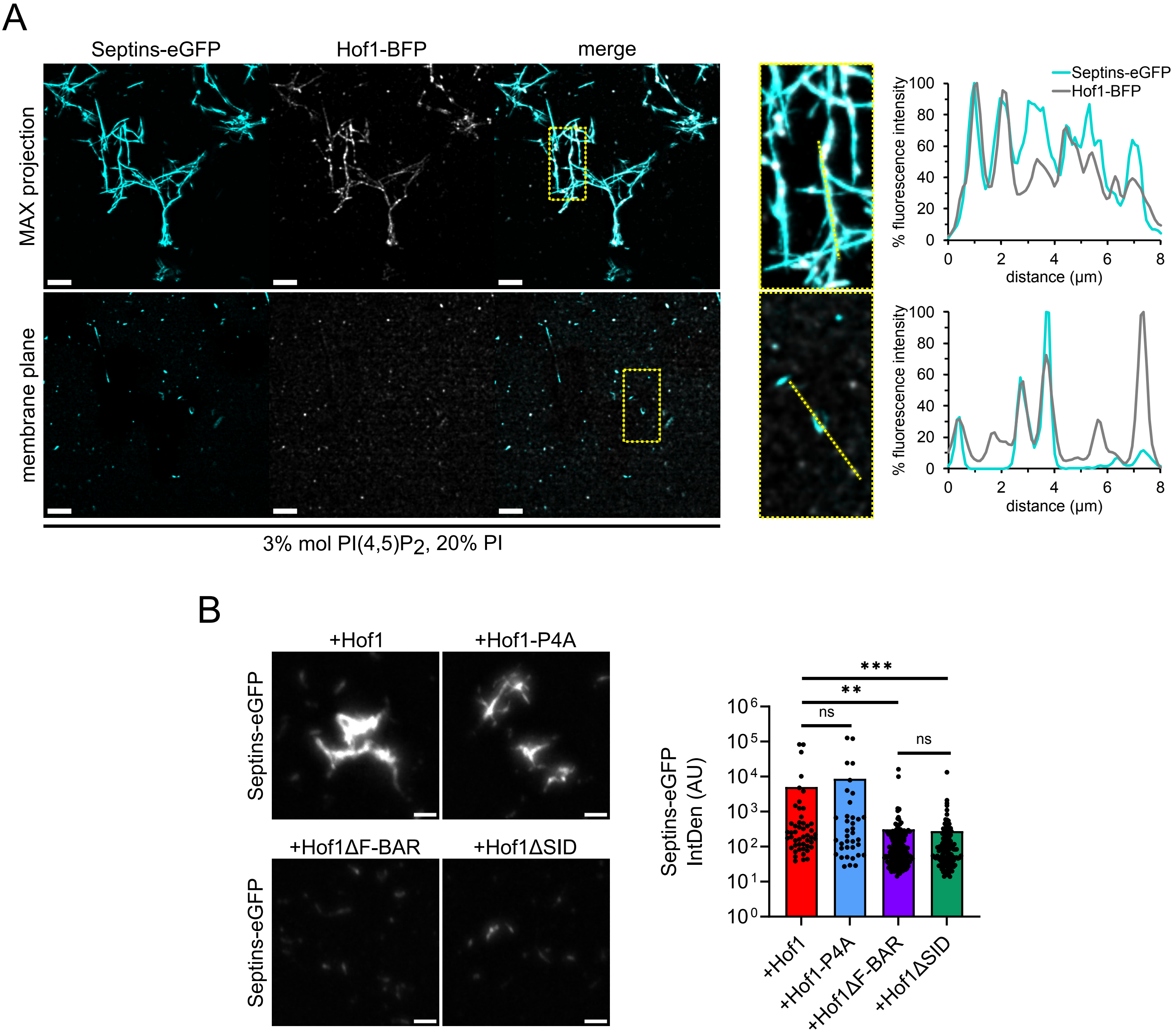
A
Septins-eGFP
Hof1-BFP
merge
MAX projection
membrane plane
Septins-eGFP
Hof1-BFP
% fluorescence intensity
distance (µm)
3% mol PI(4,5)P2, 20% PI
B
+Hof1
+Hof1-P4A
+Hof1ΔF-BAR
+Hof1ΔSID
Septins-eGFP
Septins-eGFP IntDen (AU)
ns
**
***

Figure S8. Colocalisation of Hof1-BFP with septin structures and involvement of the septin-interacting (SID) and F-BAR domains for septin organisation by Hof1 *in vitro*. **A:** A 1:1 mix of untagged and eGFP-tagged Cdc11-capped septin octamers (50 nM final concentration) were polymerised in solution by lowering the salt concentration in the absence or in the presence of two molar amounts of Hof1 that contained 20% Hof1-BFP (note that BFP tagging of Hof1 impaired its septin-bundling activity). Septin assemblies were imaged by fluorescence microscopy. Scale bar: 5μm. Magnified insets are bordered by a yellow dashed line. Fluorescence intensities in the GFP and BFP channels were measured along the dashed yellow lines and plotted on the right. **B:** A 1:1 mix of untagged and eGFP-tagged Cdc11-capped septin octamers (50 nM final concentration) were polymerised in solution by lowering the salt concentration in the absence or in the presence of equimolar amounts of the indicated Hof1 recombinant proteins. Fluorescence intensities of septin-eGFP assemblies in max-projected images (n=3) have been plotted. Scale bar: 2μm. *P*-values were calculated using an unpaired two-tailed *t*-test. Hof1 vs Hof1-P4A: ns (p=0.47; n=49 and n=37, respectively); Hof1 vs Hof1ΔF-BAR: *** (p=0.0009; n=49 and n=159, respectively); Hof1 vs Hof1ΔSID: ** (p=0.0029; n=49 and n=125, respectively); ns: not significant.

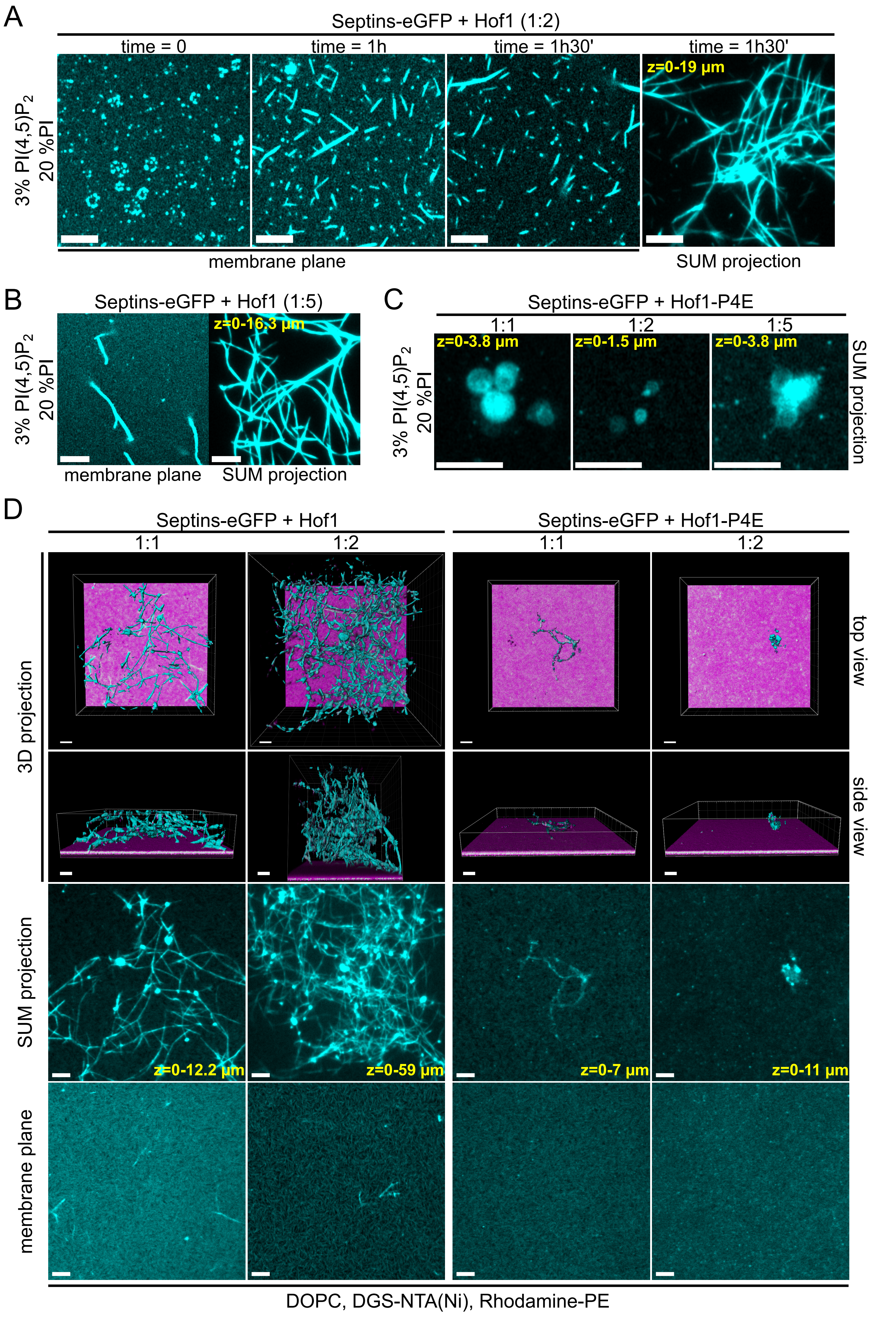
A
Septins-eGFP + Hof1 (1:2)
time = 0
time = 1h
time = 1h30'
time = 1h30'
z=0-19 µm
3% PI(4,5)P2
20 %PI
membrane plane
SUM projection
B
Septins-eGFP + Hof1 (1:5)
z=0-16.3 µm
3% PI(4,5)P2
20 %PI
membrane plane
SUM projection
C
Septins-eGFP + Hof1-P4E
1:1
1:2
1:5
z=0-3.8 µm
z=0-1.5 µm
z=0-3.8 µm
3% PI(4,5)P2
20 %PI
SUM projection
D
Septins-eGFP + Hof1
Septins-eGFP + Hof1-P4E
1:1
1:2
1:1
1:2
3D projection
top view
side view
SUM projection
z=0-12.2 µm
z=0-59 µm
z=0-7 µm
z=0-11 µm
membrane plane
DOPC, DGS-NTA(Ni), Rhodamine-PE

Figure S9. Hof1 generates septin networks on membranes.

A: A 1:1 mix of untagged and eGFP-tagged septin octamers (50 nM final concentration) was polymerised in solution by lowering the salt concentration and injected onto PI(4,5)P_2-doped SLBs. A two-fold molar amount of MPB-Hof1 was injected immediately before imaging (t=0). Images show septin structures after various times of incubation with MPB-Hof1 in different fields of view. Traces of a fluorescent lipid (TopFluor-TMR-PI(4,5)P2) were used to visualize the plane of the membrane. Septins are shown on a single membrane plane (Z=0) or sum-projected after 1h30' incubation (Z= 0-19 μm) to visualize septin structures in solution. Scale bar: 5 μm. **B:** A 1:1 mix of untagged and eGFP-tagged septin octamers (50 nM final concentration) was polymerised in the presence of 5 molar ratio of Hof1 before being injected onto SLBs doped with PI(4,5)P_2 and imaged at the membrane plane or sum-projected to visualize septin structures on top of the membrane (Z= 0-16.3 μm). Traces of a fluorescent lipid (TopFluor-TMR-PI(4,5)P2) were used to visualize the plane of the membrane. Scale bar: 5 μm. **C:** As in B) but upon incubation with different molar ratios of Hof1-4E. Sum-projected images show septin structures on top of the membrane). Scale bar: 5 μm. **D:** A 1:1 mix of untagged and eGFP-tagged septin octamers (50 nM final concentration) was polymerised in solution in the absence or presence of the indicated molar ratios of Hof1 (wild type or phospho-mimicking Hof1-P4E) before being injected onto neutral SLBs (DOPC) containing 4% DGS-NTA(Ni) lipids and imaged. Traces of a fluorescent lipid (rhodamine-PE) were used to visualize the plane of the membrane (in magenta). Z-stacks on the top of the membrane were sum-projected, while one single plane is shown at the membrane level. 3D-rendering was obtained with the Imaris software. Scale bar: 5 μm.

Table S1. List of *S. cerevisiae* strains used in this study

Name	Relevant genotype
ySP11369	*MATa, bud4::LEU2::BUD4, ura3::URA3::GFP-CDC12*
ySP11506	*MATalpha, ura3::URA3::GFP-CDC12, bud4::LEU2::BUD4, HOF1-3XminiAID::kanMX, TRP1::ADH1-OsTIR1-myc9*
ySP11951	*MATa, DMA2::GAL1-DMA2::URA3s*[a], *ura3::URA3::GFP-CDC12, bud4::LEU2::BUD4, HOF1-3XminiAID::kanMX, TRP1::ADH1-OsTIR1-myc9*
ySP11973	*MATa, DMA2::GAL1-DMA2::URA3s*[a], *bud4::LEU2::BUD4, ura3::URA3::GFP-CDC12*
ySP12439	*MATa, ura3::URA3::GFP-CDC12, bud4::LEU2::BUD4, HOF1-3XminiAID::kanMX, TRP1::ADH1-OsTIR1-myc9*
ySP12447	*MATa, bud4::LEU2::BUD4, HOF1-3XminiAID::kanMX, TRP1::ADH1-OsTIR1-myc9*
ySP12838	*MATalpha, bud4::LEU2::BUD4, ura3::URA3::GAL1-DMA2s*[a]
ySP15318	*MATalpha, bud4::LEU2::BUD4, HOF1-3XminiAID::kanMX, TRP1::ADH1-OsTIR1-myc9, ura3::URA3::GAL1-DMA2s*[a]
ySP15355	*MATalpha, bud4::LEU2::BUD4, cdc3::mCherry-CDC3::URA3*
ySP15505	*MATa, bud4::LEU2::BUD4, ura3::URA3::GAL1-DMA2m*[b], *CDC10-6Gly-yeGFP::kanMX, MOB1-2A::hphNT1*
ySP15507	*MATa, bud4::LEU2::BUD4, ura3::URA3::GAL1-DMA2m*[b], *CDC10-6Gly-yeGFP::kanMX*
ySP15648	*MATa, bud4::LEU2::BUD4, ura3::URA3::GAL1-DMA2m*[b], *CDC10-6Gly-yeGFP::kanMX, cdc15::HIS3, leu2::CDC15-HA3-7A::LEU2*
ySP15653	*MATalpha, bud4::LEU2::BUD4, ura3::URA3::GAL1-DMA2m*[b], *CDC10-6Gly-yeGFP::kanMX, MOB1-2A::hphNT1, cdc15::HIS3, leu2::CDC15-HA3-7A::LEU2*
ySP15929	*MATa, HOF1-P1E*
ySP15935	*MATa, HOF1-P2A*
ySP15971	*MATa, bud4::LEU2::BUD4, cdc3::mCherry-CDC3::URA3, HOF1-P1E*
ySP15997	*MATa, bud4::LEU2::BUD4, cdc3::mCherry-CDC3::URA3, HOF1-P2A*
ySP16026	*MATa, HOF1-P2E*
ySP16027	*MATa, HOF1-P1A*
ySP16032	*MATa, bud4::LEU2::BUD4, cdc3::mCherry-CDC3::URA3, HOF1-P2E*
ySP16038	*MATa, bud4::LEU2::BUD4, cdc3::mCherry-CDC3::URA3, HOF1-P1A*
ySP16058	*MATa, bud4::LEU2::BUD4, ura3::URA3::GAL1-DMA2m*[b], *cdc3::mCherry-CDC3::URA3*
ySP16060	*MATalpha, bud4::LEU2::BUD4, ura3::URA3::GAL1-DMA2m*[b], *cdc3::mCherry-CDC3::URA3*
ySP16089	*MATa, bud4::LEU2::BUD4, ura3::URA3::GAL1-DMA2m*[b], *cdc3::mCherry-CDC3::URA3, HOF1-P2A*
ySP16090	*MATa, bud4::LEU2::BUD4, ura3::URA3::GAL1-DMA2m*[b], *cdc3::mCherry-CDC3::URA3, HOF1-P2E*
ySP16115	*MATa, bud4::LEU2::BUD4, ura3::URA3::GAL1-DMA2m*[b], *cdc3::mCherry-CDC3::URA3, HOF1-P1E*
ySP16136	*MATa, bud4::LEU2::BUD4, cdc3::mCherry-CDC3::URA3, shs1::natNT2*
ySP16137	MATa, *bud4::LEU2::BUD4, cdc3::mCherry-CDC3::URA3, shs1::natNT2, HOF1-P1A*
ySP16139	*MATa, bud4::LEU2::BUD4, cdc3::mCherry-CDC3::URA3, shs1::natNT2, HOF1-P1E*
ySP16141	*MATa, bud4::LEU2::BUD4, cdc3::mCherry-CDC3::URA3, shs1::natNT2, HOF1-P2A*

ySP16143	*MATa, bud4::LEU2::BUD4, cdc3::mCherry-CDC3::URA3, shs1::natNT2, HOF1-P2E*
ySP16154	*MATa, bud4::LEU2::BUD4, ura3::URA3::GAL1-DMA2m*[b]*, cdc3::mCherry-CDC3::URA3, HOF1-P2E*
ySP16166	*MATalpha, bud4::LEU2::BUD4, trp1::TRP1::CDC14*$^{TAB6-1}$*, cdc15::CDC15-as1(L99G)::URA3, cdc3::mCherry-CDC3, HOF1-P1A*
ySP16169	*MATalpha, bud4::LEU2::BUD4, trp1::TRP1::CDC14*$^{TAB6-1}$*, cdc15::CDC15-as1(L99G)::URA3, cdc3::mCherry-CDC3*
ySP16170	*MATa, bud4::LEU2::BUD4, trp1::TRP1::CDC14*$^{TAB6-1}$*, cdc15::CDC15-as1(L99G)::URA3, cdc3::mCherry-CDC3, HOF1-P1E*
ySP16211	*MATa, bud4::LEU2::BUD4, ura3::URA3::GAL1-DMA2m*[b]*, cdc3::mCherry-CDC3::URA3, HOF1-P1A*
ySP16253	*MATa, bud4::LEU2::BUD4, trp1::TRP1::CDC14*$^{TAB6-1}$*, cdc15::CDC15-as1(L99G)::URA3, cdc3::mCherry-CDC3, HOF1-P2A*
ySP16260	*MATa, HOF1-P3A*
ySP16288	*MATa, HOF1-P3E*
ySP16357	*MATa, bud4::LEU2::BUD4, cdc3::mCherry-CDC3::URA3, HOF1-3XminiAID::kanMX, TRP1::ADH1-OsTIR1-myc9*
ySP16363	*MATa, HOF1-P2E, bud4::LEU2::BUD4, trp1::TRP1::CDC14*$^{TAB6-1}$*, cdc15::CDC15-as1(L99G)::URA3, cdc3::mCherry-CDC3*
ySP16469	*MATa, HOF1-P4E*
ySP16470	*MATa, HOF1-P4A*
ySP16488	*MATa, bud4::LEU2::BUD4, ura3::URA3::GAL1-DMA2m*[b]*, cdc3::mCherry-CDC3::URA3, HOF1-P4E::K.l.TRP1::RCH1*
ySP16502	*MATa, bud4::LEU2::BUD4, ura3::URA3::GAL1- DMA2m*[b]*, cdc3::mCherry-CDC3::URA3, HOF1-P3A::K.l.TRP1::RCH1*
ySP16544	*MATa, bud4::LEU2::BUD4, cdc3::mCherry-CDC3::URA3, HOF1-P3E-eGFP::kanMX4*
ySP16551	*MATa, bud4::LEU2::BUD4, ura3::URA3::GAL1-DMA2m*[b]*, cdc3::mCherry-CDC3::URA3, HOF1-P3E*
ySP16568	*MATalpha, bud4::LEU2::BUD4, ura3::URA3::GAL1-DMA2m*[b]*, cdc3::mCherry-CDC3::URA3, HOF1-P4A*
ySP16600	*MATa, bud4::LEU2::BUD4, cdc3::mCherry-CDC3::URA3, HOF1-P2A-eGFP::kanMX4*
ySP16605	*MATa, bud4::LEU2::BUD4, cdc3::mCherry-CDC3::URA3, HOF1-P2E-eGFP::kanMX4*
ySP16610	*MATa, bud4::LEU2::BUD4, cdc3::mCherry-CDC3::URA3, HOF1-P1A-eGFP::kanMX4*
ySP16612	*MATa, bud4::LEU2::BUD4, cdc3::mCherry-CDC3::URA3, HOF1-P1E-eGFP::kanMX4*
ySP16637	*MATalpha, bud4::LEU2::BUD4, cdc3::mCherry-CDC3::URA3, HOF1-P3A-eGFP::kanMX4*
ySP16639	*MATa, bud4::LEU2::BUD4, cdc3::mCherry-CDC3::URA3, HOF1-P4E-eGFP::kanMX4*
ySP16727	*MATalpha, bud4::LEU2::BUD4, cdc3::mCherry-CDC3::URA3, ura3::URA3::GAL1-DMA2s*[a]
ySP16728	*MATa, HOF1-P5A*
ySP16736	*MATa, bud4::LEU2::BUD4, cdc3::mCherry-CDC3::URA3*
ySP16739	*MATa, bud4::LEU2::BUD4, HOF1-P4A-3HA::K.l.URA3*
ySP16742	*MATa, bud4::LEU2::BUD4, cdc3::mCherry-CDC3::URA3, HOF1-P4A-eGFP::kanMX4*
ySP16743	*MATa, HOF1-P5E*
ySP16765	*MATa, bud4::LEU2::BUD4, ura3::URA3::GAL1-DMA2m*[b]*, cdc3::mCherry-CDC3::URA3, HOF1-P4A::K.l.TRP1::RCH1*
ySP16782	*MATa, bud4::LEU2::BUD4, cdc3::mCherry-CDC3::URA3, HOF1-eGFP::kanMX4*
ySP16821	*MATalpha, bud4::LEU2::BUD4, ura3::URA3::GAL1-DMA2m*[b]*, cdc3::mCherry-CDC3::URA3, HOF1-P5A*
ySP16823	*MATa, bud4::LEU2::BUD4, ura3::URA3::GAL1-DMA2m*[b]*, cdc3::mCherry-CDC3::URA3, HOF1-P5E*
ySP16852	*MATalpha, bud4::LEU2::BUD4, HOF1-P1A-3HA::K.l.URA3*
ySP16856	*MATa, bud4::LEU2::BUD4, HOF1-P1E-3HA::K.l.URA3*
ySP16902	*MATa, bud4::LEU2::BUD4, HOF1-P4E-3HA::K.l.URA3*

ySP16910	*MATa, bud4::LEU2::BUD4, cdc3::mCherry-CDC3::URA3, HOF1(ΔF-BAR)-P4E*
ySP16925	*MATa, bud4::LEU2::BUD4, HOF1-3HA::K.l.URA3*
ySP16928	*MATa, bud4::LEU2::BUD4, ura3::URA3::GAL1-DMA2m*[b]*, cdc3::mCherry-CDC3::URA3, HOF1(ΔF-BAR)*
ySP16931	*MATa, bud4::LEU2::BUD4, cdc3::mCherry-CDC3::URA3, HOF1(ΔF-BAR)*
ySP16956	*MATa, bud4::LEU2::BUD4, CDC10-6xGly-3xFLAG-kanMX6, HOF1-P1A-3HA::K.l.URA3*
ySP16958	*MATa, bud4::LEU2::BUD4, CDC10-6xGly-3xFLAG-kanMX6, HOF1-P1E-3HA::K.l.URA3*
ySP16964	*MATa, bud4::LEU2::BUD4, CDC10-6xGly-3xFLAG-kanMX6, HOF1-P4A-3HA::K.l.URA3*
ySP16966	*MATa, bud4::LEU2::BUD4, CDC10-6xGly-3xFLAG-kanMX6, HOF1-3HA::K.l.URA3*
ySP16971	*MATa, bud4::LEU2::BUD4, ura3::URA3::GAL1-DMA2m*[b]*, cdc3::mCherry-CDC3::URA3, HOF1(ΔF-BAR)-P2E*
ySP16973	*MATa, bud4::LEU2::BUD4, ura3::URA3::GAL1-DMA2m*[b]*, cdc3::mCherry-CDC3::URA3, HOF1(ΔF-BAR)-P1E*
ySP16976	*MATa, bud4::LEU2::BUD4, cdc3::mCherry-CDC3::URA3, HOF1(ΔF-BAR)-P2E*
ySP16977	*MATalpha, bud4::LEU2::BUD4, cdc3::mCherry-CDC3::URA3, HOF1(ΔF-BAR)-P1E*
ySP16997	*MATa, bud4::LEU2::BUD4, trp1::TRP1::CDC14*$^{TAB6-1}$*, cdc15::CDC15-as1(L99G)::URA3, cdc3::mCherry-CDC3, HOF1-P4E*
ySP17059	*MATa, bud4::LEU2::BUD4, trp1::TRP1::CDC14*$^{TAB6-1}$*, cdc15::CDC15-as1(L99G)::URA3, cdc3::mCherry-CDC3, HOF1-P4A*
ySP17087	*MATa, bud4::LEU2::BUD4, ura3::URA3::GAL1-DMA2m*[b]*, cdc3::mCherry-CDC3::URA3, HOF1(ΔF-BAR)-P4E*
ySP17093	*MATalpha, bud4::LEU2::BUD4, cdc3::mCherry-CDC3::URA3, HOF1(ΔF-BAR)-P1E-eGFP::kanMX4*
ySP17096	*MATa, bud4::LEU2::BUD4, cdc3::mCherry-CDC3::URA3, (HOF1ΔF-BAR)-P4E-eGFP::kanMX4*
ySP17097	*MATa, bud4::LEU2::BUD4, cdc3::mCherry-CDC3::URA3, HOF1(ΔF-BAR)-eGFP:kanMX4*
ySP17099	*MATa, bud4::LEU2::BUD4, cdc3::mCherry-CDC3::URA, HOF1(ΔF-BAR)-P2E-eGFP::kanMX4*
ySP17266	*MATa/MATalpha, HOF1/hof1::natNT2*
ySP17278	*MATa, hof1::natNT2*
ySP17279	*MATa, hof1::natNT2*
ySP17280	*MATalpha, hof1::natNT2*
ySP17281	*MATalpha, hof1::natNT2*
ySP17377	*MATalpha bud4::LEU2::BUD4, cdc3::mCherry-CDC3::URA3, ura3::URA3::GAL1-DMA2s*[a]*, hof1::natNT2,*
ySP17378	*MATa, bud4::LEU2::BUD4, cdc3::mCherry-CDC3::URA3, ura3::URA3::GAL1-DMA2s*[a]*, hof1::natNT2*
ySP17382	*MATalpha, bud4::LEU2::BUD4, cdc3::mCherry-CDC3::URA3, hof1::natNT2*
ySP17385	*MATa, cdc3::mCherry-CDC3::URA3, hof1::natNT2*
ySP17525	*MATalpha, bud4::LEU2::BUD4, CDC10-6xGly-3xFLAG-kanMX6, HOF1-3HA::KlURA3*
ySP17777	*MATa, bud4::LEU2::BUD4, HOF1-3HA::K.l.URA3, ura3::URA3::GAL1-DMA2m*[b]
ySP17779	*MATa, bud4::LEU2::BUD4, HOF1-3HA::K.l.URA3, dbf20::natN2, dbf2-2*
ySP17835	*MATa, HOF1(F-BAR1), bud4::LEU2::BUD4, cdc3::mCherry-CDC3::URA3*
ySP17841	*MATa, HOF1(F-BAR2), bud4::LEU2::BUD4, cdc3::mCherry-CDC3::URA3*
ySP17848	*MATa, HOF1(F-BAR3), bud4::LEU2::BUD4, cdc3::mCherry-CDC3::URA3*
ySP17887	*MATa, bud4::LEU2::BUD4, HOF1-P1A-3HA::K.l.URA3*
ySP17888	*MATa, HOF1(F-BAR2)-P4E, bud4::LEU2::BUD4, ura3::URA3::GAL1-DMA2m*[b]*, cdc3::mCherry-CDC3::URA3*
ySP17892	*MATa, HOF1(F-BAR1)-P4E, bud4::LEU2::BUD4, ura3::URA3::GAL1-DMA2m*[b]*, cdc3::mCherry-CDC3::URA3*

ySP17915 *MATalpha, HOF1(F-BAR3)-P4E, bud4::LEU2::BUD4, ura3::URA3::GAL1-DMA2m[b], cdc3::mCherry-CDC3::URA3*
ySP17907 *MATa, bud4::LEU2::BUD4, HOF1-3HA::K.l.URA3, grr1-17*
ySP17909 *MATa, bud4::LEU2::BUD4, HOF1-P4A-3HA::K.l.URA3, grr1-17*
ySP17911 *MATa, bud4::LEU2::BUD4, HOF1-P4E-3HA::K.l.URA3, grr1-17*

[a]: all strains marked *GAL1-DMA2s* carry one single copy of the *GAL1-DMA2* construct integrated in the genome
[b]: all strains marked *GAL1-DMA2m* carry multiple copies (≥3) of the *GAL1-DMA2* construct integrated in the genome. All these strains are derived by genetic crosses and therefore carry the same copy number of integrated *GAL1-DMA2.*

The mutated residues in the *HOF1* mutants are the following:

HOF1-P1A: S313A-S314A-S337A-T341A-T350A-S366A-S369A-S370A-S421A-S423A-S424A-S478A-S563A
HOF1-P1E: S313E-S314E-S337E-T341E-T350E-S366E-S369E-S370E-S421E-S423E-S424E-S478E-S563E
HOF1-P2A: S313A-T350A-T357A-S421A-S478A-S480A-T516A-S517A-T518A-S519A-T520A-S563A
HOF1-P2E: S313E-T350E-T357E-S421E-S478E-S480E-T516E-S517E-T518E-S519E-T520E-S563E
HOF1-P3A: S337A-T341A-T350A-S349A-T364A-S366A-S369A-S370A-S381A-S423A-S424A-S437A-T450A-T498A-T516A-S517A-T518A-S519A-T520A
HOF1-P3E: S337E-T341E-T350E-S349E-T364E-S366E-S369E-S370E-S381E-S423E-S424E-S437E-T450E-T498E-T516E-S517E-T518E-S519E-T520E
HOF1-P4A: S313A-S314A-S337A-T341A-T350A-S366A-S421A-S423A-S424A-S563A
HOF1-P4E: S313E-S314E-S337E-T341E-T350E-S366E-S421E-S423E-S424E-S563E
HOF1-P5A: S369A-S370A-S478A
HOF1-P5E: S369E-S370E-S478E
HOF1(ΔF-BAR) Δ2-279
HOF1(F-BAR1) K39E-R41E-K58E
HOF1(F-BAR2) K46E-R50E-R51E
HOF1(F-BAR3) R99E-K106E

Table S2. List of primers used in this study for gene tagging, amplifying repair DNA for genome editing, and cloning

Sequences in bold anneal to the tag-bearing cassette

MP434 (tagging *CDC10* with *6xGly-3xFLAG-kanMX6*; fwd)

ATGCGAATAGTCGTTCCTCAGCTCATATGTCTAGCAACGCCATTCAACGT**GGGGGAGGCGGGGGTGGA**

MP435 (tagging *CDC10* with *6xGly-3xFLAG-kanMX6*; rev)

GAGAATTCTTAATAACATAAGATATATAATCACCACCATTCTTATGAGAT**GAATTCGAGCTCGTTTAAAC**

MP443 (tagging *HOF1* and *HOF1* mutants with *eGFP::kanMX*; fwd)

AGGATAGGATTAATTCCCTATAATTTCATTCAGCTACTGCATCAAGGTCTT**CGTACGCTGCAGGTCGAC**

MP444 (deletion of *HOF1* with *natNT2* and tagging *HOF1* and *HOF1* mutants with *eGFP::kanMX*; rev)

ATACTTTTTTTCTTTTATCAGAAAACTAGTAAAATTGATATACATCGAGA**ATCGATGAATTCGAGCTCG**

MP467 (deletion of *HOF1* with *natNT2*; fwd)

GGTGAAAGAATGAGCTACAGTTATGAAGCTTGTTTTTGGGACCCAAACGAC**CGTACGCTGCAGGTCGAC**

MP1130 (tagging *HOF1* and *HOF1* mutants with *3HA::KlURA3*; fwd)

AGGATAGGATTAATTCCCTATAATTTCATTCAGCTACTGCATCAAGGTCTT**TCCGGTTCTGCTGCTAG**

MP1131 (tagging *HOF1* and *HOF1* mutants with *3HA::KlURA3*; rev)

TATACTTTTTTTCTTTTATCAGAAAACTAGTAAAATTGATATACATCGAGA**CCTCGAGGCCAGAAGAC**

MP1183 (introducing *kanMX4* at the 3'UTR of *HOF1*; fwd)

TAGGATTAATTCCCTATAATTTCATTCAGCTACTGCATCAAGGTCTT<u>TGA</u>**CGTACGCTGCAGGTCGAC**

MP1184 (introducing *kanMX4* at the 3'UTR of *HOF1*; rev)

ATACTTTTTTTCTTTTATCAGAAAACTAGTAAAATTGATATACATCGAGA**ATCGATGAATTCGAGCTCG**

MP1218 (for cloning Hof1-P4E; rev)

GTAATACCTCTATCCCTGACC

MP1257 (for *HOF-P1A, HOF1-P2E, HOF1-P3A, HOF1-P3E, HOF1-P4A* and *HOF1-P4E* genome editing and for cloning *HOF1-P4E*; Fwd)

CACGGCAGCGGATGAAATATC

MP1258 (for *HOF1-P1A, HOF1-P2E, HOF1-P3A, HOF1-P3E, HOF1-P4A, HOF1-P4E, HOF1-P5A* and *HOF1-P5E* genome editing; Rev)

CACGGCAGCGGATGAAATATC

MP1301 (tagging *RCH1* with *K.l.TRP1*; fwd)

AAAATCAATCCGTTCTGTCGAACAACGTCCCATATACACAACCAAGGTGA**GCTAACGTCTTGCCAGATG**

MP1302 (tagging *RCH1* with *K.l.TRP1*; rev)

ATAAACGTTATGTAATATATAGAATTCATGCAAAATTAACAAGGGAAATA**CGTCGACCTCGAGGCCAGAA**

MP1362 (*HOF1-P5A* and *HOF1-P5E* genome editing; Fwd)

CCATTGCCATCTCCAGAAGTC

MP1394 (introducing *kanMX4* at the 5'UTR of *HOF1*; fwd)

CGAGGGTGCCCCTTTCGGTAGTTTGCGATTTTCTTCCAGAAGAGCACCGA**CGTACGCTGCAGGTCGAC**

MP1395 (introducing *kanMX4* at the 5'UTR of *HOF1*; rev)

TTCGGTCCGCTGTTACCCAAAAGGGAAATAGTACTTTCCGGATGGGGCAA**ATCGATGAATTCGAGCTCG**

MP1396 (genome editing of *HOF1(∆F-BAR*); fwd)

ACTGCAAGCAACAAGGAGTTC

MP1397 (genome editing of *HOF1(∆F-BAR*); rev)

CCATGGTGACTTCTGGAGATG

MP1429 (genome editing of *HOF1(F-BAR)* mutants by; fwd)

ATAAACATTGGTGATATACTG

MP1430 (genome editing of *HOF1(ΔF-BAR*) in phosphomimetic mutant by genome editing; rev)

AGTTTCCTTATATTGTCCATA

MP1444 (cloning of *HOF1-P4A* and *HOF1-P4E*; Fwd)

CATCATGGATGAGAGAGGTG;

MP1454 (cloning of *HOF1-P4A* and *HOF1-P4E*; Rev)

GTTTCAGTCTACCAGTTCC

MP1492 (cloning of *HOF1-BFP* by Gibson assembly; vector, Fwd)

CATCATCACCATCACCACTG

MP1493 (cloning of *HOF1-BFP* by Gibson assembly; vector, Rev)

AAGACCTTGATGCAGTAGCT

MP1495 (cloning of *HOF1-BFP* by Gibson assembly; insert, Rev)

AGTGGTGATGGTGATGATGattgagcttgtgccccagtttg

MP1597 (deleting F-BAR domain in MBP-Hof1-6His expression plasmid pBG2163; Fwd)

tcagaattcggatccATGAAAGGTGACATGAATTCTAGC

MP1598 (deleting F-BAR domain in MBP-Hof1-6His expression plasmid pBG2163; Rev)

GAATTCATGTCACCTTTCATggatccgaattctgaaatc

MP1599 (deleting SID domain in MBP-Hof1-6His expression plasmid pBG2163; Fwd)

ccaactgggctcaaatgGAGACAAAAGTGGTTGCTAACC

MP1600 (deleting SID domain in MBP-Hof1-6His expression plasmid pBG2163; Rev)

TTAGCAACCACTTTTGTCTCcatttgagcccagttggc

MP1604 (genome editing of *HOF1(F-BAR1), HOF1(F-BAR2), HOF1(F-BAR1)-P4E* and *HOF1(F-BAR2)-P4E*; Rev)

TAGTATCCGTGTAAATCTGTC

MP1605 (genome editing of *HOF1(F-BAR3)* and *HOF1(F-BAR3)-P4E*; Fwd)

CAAGTCTCTGAATCTAATCTG

MP1614 (cloning of *HOF1-BFP* by Gibson assembly; insert, Fwd)

AGCTACTGCATCAAGGTCTTagcgagctgattaaggagaac

Table S3. Plasmids used in this study for genome editing

pSP1648 (pTwist Amp^r *HOF1-P1A* fragment (1264 bp before stop codon (position1254 in CDS) to 121 bp in 3'UTR)

AA substitutions, 313:S->A; 314:S->A; 337:S->A; 341:T->A; 350:T->A; 366:S->A; 369:S->A; 370:S->A; 421:S->A; 423:S->A; 424:S->A; 478:S->A; 563:S->A.

Codon substitutions, 937:TCT->GCT; 940:TCT->GCT; 1009:TCT->GCT; 1021:ACC->GCT; 1048:ACA->GCT; 1096:TCT->GCT; 1105:TCT->GCT; 1108:TCA->GCT; 1261:TCT->GCT; 1267:AGC->GCT; 1270:TCA->GCT; 1432:TCT->GCT; 1687:AGT->GCT.

GTTAACAAATCATCTGAACTCATTCACGGCAGCGGATGAAATATCGACGTTTTCAAAAGAAAATGGAACTGGTAGACTGAAACATAAGACTT
CCAAAGGTGACATGAATTCTAGCGCCAACTGGGCTCAAATGAGCAGTATCTCCACGACAAGTAAAAAAACCGAATCGTATATGGACAATATA
AGGAAACTAGCTGCTCAGCTGAAGGAAACCGAAAATAAAAGGAAACTAGCTTCAATAGACAAATATGAGAAACCATTGCCAGCTCCAGAAGT
CGCTATGGCTACACAATTTAGAAATTCCGCTCCCGTCATACGTAATGAGACAAAAGTGGTTGCTAACCCAACATTAGCTTTGAGGGCTGCTC
CCGTCCAGTTACAGAGCAATGTGGATGACTCCGTGTTAAGGCAGAAACCTGATAAACCAAGGCCGATTGTTGGGGAAGAACAACTTAAACC
TGATGAAGATTCGAAAAATCCTGATGAAAAAGGTCTAATGGTGCATAAAAGAAATCAAGCTCTCGCTGCTCCATCAGAATCAAGTTCTTCTAA

TCCAACGGATTTTAGCCACATCAAAAAGAGACAAAGTATGGAATCTATGACTACATCCGTAAGTTCAATGGCCAATAGTATAGACGACTCAC
AGAGATTTGCCAAATCATGGAACTCGTCAAATAGGAAAAGAAAAGCTATGAGCCATTTGCAAGTACCCTCATCCGCTTCTTCGAGGTCGGAT
GATGGGGGAAGAACGCCTAACTCTGCGCACAACCTCAATGAAGATGATTATAACACGAGGAGAGATACCAGTACAAGCACAATTTTGTTCA
AACCTCCTGTGGCAGTAAGAGGAACGTCTAGGGGGCACACGCATAGACAATCCATGATAATGCAAGATTCAAGTAATCCAATTGAAGATGC
CTTGTACGAAATGGAAAGAATCCAAGCTAGTTCTAAACCAGGTACAAAAACAGGAAACATCATGGATGAGAGAGGTGTGGTCAGGGATAGA
GGTATTACTGTTACCTTGCCTATTGTCACCAGTGAAGGTTTTCCAGTCATTGAATATGCCAAGGCCATGTATCCATTGATTGGAAATGAGGC
ACCTGGATTAGCGAATTTCCATAAGGGCGACTATCTGCTAATTACTGAGATTGTTAATAAAGATTGGTATAAAGGTGAAGTTTATGATAATGA
CCGTATAGATAGAAATCATAGGATAGGATTAATTCCCTATAATTTCATTCAGCTACTGCATCAAGGTCTTTGATCTCGATGTATATCAATTTTA
CTAGTTTTCTGATAAAAGAAAAAAAGTATAATGTCAGATTTTTGTTAAGTAGAAATAGAAAGATATACGCTGCCGTGGGAGAAGGACACCTCT
CCGG

pSP1673 (pTwist Amp^r *HOF1-P1E* fragment (1264 bp before stop codon (position1254 in CDS) to 121 bp in 3'UTR)

AA substitutions, 313:S->E; 314:S->E; 337:S->E; 341:T->E; 350:T->E; 366:S->E; 369:S->E; 370:S->E;

421:S->E; 423:S->E; 424:S->E; 478:S->E; 563:S->E.

Codon substitution, 937:TCT->GAA; 940:TCT->GAA; 1009:TCT->GAA; 1021:ACC->GAA; 1048:ACA->GAA; 1096:TCT->GAA; 1105:TCT->GAA; 1108:TCA->GAA; 1261:TCT->GAA; 1267:AGC->GAG; 1270:TCA->GAA; 1432:TCT->GAA; 1687:AGT->GAA.

GTTAACAAATCATCTGAACTCATTCACGGCAGCGGATGAAATATCGACGTTTTCAAAAGAAAATGGAACTGGTAGACTGAAACATAAGACTT
CCAAAGGTGACATGAATTCTAGCGCCAACTGGGCTCAAATGAGCAGTATCTCCACGACAAGTAAAAAAACCGAATCGTATATGGACAATATA
AGGAAACTAGAAGAACAGCTGAAGGAAACCGAAAATAAAAGGAAACTAGCTTCAATAGACAAATATGAGAAACCATTGCCAGAACCAGAAGT
CGAAATGGCTACACAATTTAGAAATTCCGAACCCGTCATACGTAATGAGACAAAAGTGGTTGCTAACCCAACATTAGAATTGAGGGAAGAAC
CCGTCCAGTTACAGAGCAATGTGGATGACTCCGTGTTAAGGCAGAAACCTGATAAACCAAGGCCGATTGTTGGGGAAGAACAACTTAAACC
TGATGAAGATTCGAAAAATCCTGATGAAAAAGGTCTAATGGTGCATAAAAGAAATCAAGAACTCGAGGAACCATCAGAATCAAGTTCTTCTAA
TCCAACGGATTTTAGCCACATCAAAAAGAGACAAAGTATGGAATCTATGACTACATCCGTAAGTTCAATGGCCAATAGTATAGACGACTCAC
AGAGATTTGCCAAATCATGGAACTCGTCAAATAGGAAAAGAAAAGAAATGAGCCATTTGCAAGTACCCTCATCCGCTTCTTCGAGGTCGGAT
GATGGGGGAAGAACGCCTAACTCTGCGCACAACCTCAATGAAGATGATTATAACACGAGGAGAGATACCAGTACAAGCACAATTTTGTTCA
AACCTCCTGTGGCAGTAAGAGGAACGTCTAGGGGGCACACGCATAGACAATCCATGATAATGCAAGATTCAAGTAATCCAATTGAAGATGC
CTTGTACGAAATGGAAAGAATCCAAGAAAGTTCTAAACCAGGTACAAAAACAGGAAACATCATGGATGAGAGAGGTGTGGTCAGGGATAGA
GGTATTACTGTTACCTTGCCTATTGTCACCAGTGAAGGTTTTCCAGTCATTGAATATGCCAAGGCCATGTATCCATTGATTGGAAATGAGGC
ACCTGGATTAGCGAATTTCCATAAGGGCGACTATCTGCTAATTACTGAGATTGTTAATAAAGATTGGTATAAAGGTGAAGTTTATGATAATGA
CCGTATAGATAGAAATCATAGGATAGGATTAATTCCCTATAATTTCATTCAGCTACTGCATCAAGGTCTTTGATCTCGATGTATATCAATTTTA

CTAGTTTTCTGATAAAAGAAAAAAAGTATAATGTCAGATTTTTGTTAAGTAGAAATAGAAAGATATACGCTGCCGTGGGAGAAGGACACCTCT
CCGG

pSP1674 (pTwist Amp^r *HOF1-P2A* fragment (1264 bp before stop codon (position1254 in CDS) to 121 bp in 3'UTR)

AA substitutions, 313:S->A; 350:T->A; 357:T->A; 421:S->A; 478:S->A; 480:S->A; 516:T->A; 517:S->A; 518:T->A; 519:S->A; 520:T->A; 563:S->A.

Codon substitutions, 937:TCT->GCT; 1048:ACA->GCT; 1069:ACA->GCT; 1261:TCT->GCT; 1432:TCT->GCT; 1438:AGC->GCT; 1546:ACC->GCT; 1549:AGT->GCA; 1552:ACA->GCT; 1555:AGC->GCT; 1558:ACA->GCT; 1687:AGT->GCT.

GTTAACAAATCATCTGAACTCATTCACGGCAGCGGATGAAATATCGACGTTTTCAAAAGAAAATGGAACTGGTAGACTGAAACATAAGACTT
CCAAAGGTGACATGAATTCTAGCGCCAACTGGGCTCAAATGAGCAGTATCTCCACGACAAGTAAAAAAACCGAATCGTATATGGACAATATA
AGGAAACTAGCTTCTCAGCTGAAGGAAACCGAAAATAAAAGGAAACTAGCTTCAATAGACAAATATGAGAAACCATTGCCATCTCCAGAAGT
CACCATGGCTACACAATTTAGAAATTCCGCTCCCGTCATACGTAATGAGGCTAAAGTGGTTGCTAACCCAACATTATCTTTGAGGTCTTCAC
CCGTCCAGTTACAGAGCAATGTGGATGACTCCGTGTTAAGGCAGAAACCTGATAAACCAAGGCCGATTGTTGGGGAAGAACAACTTAAACC
TGATGAAGATTCGAAAAATCCTGATGAAAAAGGTCTAATGGTGCATAAAAGAAATCAAGCTCTCAGCTCACCATCAGAATCAAGTTCTTCTAA
TCCAACGGATTTTAGCCACATCAAAAAGAGACAAAGTATGGAATCTATGACTACATCCGTAAGTTCAATGGCCAATAGTATAGACGACTCAC
AGAGATTTGCCAAATCATGGAACTCGTCAAATAGGAAAAGAAAAGCTATGGCTCATTTGCAAGTACCCTCATCCGCTTCTTCGAGGTCGGAT
GATGGGGGAAGAACGCCTAACTCTGCGCACAACCTCAATGAAGATGATTATAACACGAGGAGAGATGCTGCAGCTGCTGCTATTTTGTTCA
AACCTCCTGTGGCAGTAAGAGGAACGTCTAGGGGGCACACGCATAGACAATCCATGATAATGCAAGATTCAAGTAATCCAATTGAAGATGC
CTTGTACGAAATGGAAAGAATCCAAGCTAGTTCTAAACCAGGTACAAAAACAGGAAACATCATGGATGAGAGAGGTGTGGTCAGGGATAGA
GGTATTACTGTTACCTTGCCTATTGTCACCAGTGAAGGTTTTCCAGTCATTGAATATGCCAAGGCCATGTATCCATTGATTGGAAATGAGGC
ACCTGGATTAGCGAATTTCCATAAGGGCGACTATCTGCTAATTACTGAGATTGTTAATAAAGATTGGTATAAAGGTGAAGTTTATGATAATGA
CCGTATAGATAGAAATCATAGGATAGGATTAATTCCCTATAATTTCATTCAGCTACTGCATCAAGGTCTTTGATCTCGATGTATATCAATTTTA
CTAGTTTTCTGATAAAAGAAAAAAAGTATAATGTCAGATTTTTGTTAAGTAGAAATAGAAAGATATACGCTGCCGTGGGAGAAGGACACCTCT
CCGG

pSP1651 (pTwist Amp^r *HOF1-P2E* fragment (1264 bp before stop codon (position1254 in CDS) to 121 bp in 3'UTR)

AA substitutions, 313:S->E; 350:T->E; 357:T->E; 421:S->E; 478:S->E; 480:S->E; 516:T->E; 517:S->E; 518:T->E; 519:S->E; 520:T->E; 563:S->E.

Codon substitutions, 937:TCT->GAA; 1048:ACA->GAA; 1069:ACA->GAG; 1261:TCT->GAG; 1432:TCT->GAA; 1438:AGC->GAA; 1546:ACC->GAA; 1549:AGT->GAA; 1552:ACA->GAA; 1555:AGC->GAA; 1558:ACA->GAA; 1687:AGT->GAA.

GTTAACAAATCATCTGAACTCATTCACGGCAGCGGATGAAATATCGACGTTTTCAAAAGAAAATGGAACTGGTAGACTGAAACATAAGACTT
CCAAAGGTGACATGAATTCTAGCGCCAACTGGGCTCAAATGAGCAGTATCTCCACGACAAGTAAAAAAACCGAATCGTATATGGACAATATA
AGGAAACTAGAATCTCAGCTGAAGGAAACCGAAAATAAAAGGAAACTAGCTTCAATAGACAAATATGAGAAACCATTGCCATCTCCAGAAGT
CACCATGGCTACACAATTTAGAAATTCCGAACCCGTCATACGTAATGAGGAGAAAGTGGTTGCTAACCCAACATTATCTTTGAGGTCTTCAC
CCGTCCAGTTACAGAGCAATGTGGATGACTCCGTGTTAAGGCAGAAACCTGATAAACCAAGGCCGATTGTTGGGGAAGAACAACTTAAACC
TGATGAAGATTCGAAAAATCCTGATGAAAAAGGTCTAATGGTGCATAAAAGAAATCAAGAGCTCAGCTCACCATCAGAATCAAGTTCTTCTAA
TCCAACGGATTTTAGCCACATCAAAAAGAGACAAAGTATGGAATCTATGACTACATCCGTAAGTTCAATGGCCAATAGTATAGACGACTCAC
AGAGATTTGCCAAATCATGGAACTCGTCAAATAGGAAAAGAAAAGAAATGGAACATTTGCAAGTACCCTCATCCGCTTCTTCGAGGTCGGAT
GATGGGGGAAGAACGCCTAACTCTGCGCACAACCTCAATGAAGATGATTATAACACGAGGAGAGATGAAGAAGAAGAAGAAATTTTGTTCA
AACCTCCTGTGGCAGTAAGAGGAACGTCTAGGGGGCACACGCATAGACAATCCATGATAATGCAAGATTCAAGTAATCCAATTGAAGATGC
CTTGTACGAAATGGAAAGAATCCAAGAAAGTTCTAAACCAGGTACAAAAACAGGAAACATCATGGATGAGAGAGGTGTGGTCAGGGATAGA
GGTATTACTGTTACCTTGCCTATTGTCACCAGTGAAGGTTTTCCAGTCATTGAATATGCCAAGGCCATGTATCCATTGATTGGAAATGAGGC
ACCTGGATTAGCGAATTTCCATAAGGGCGACTATCTGCTAATTACTGAGATTGTTAATAAAGATTGGTATAAAGGTGAAGTTTATGATAATGA
CCGTATAGATAGAAATCATAGGATAGGATTAATTCCCTATAATTTCATTCAGCTACTGCATCAAGGTCTTTGATCTCGATGTATATCAATTTTA
CTAGTTTTCTGATAAAAGAAAAAAAGTATAATGTCAGATTTTTGTTAAGTAGAAATAGAAAGATATACGCTGCCGTGGGAGAAGGACACCTCT
CCGG

pSP1699 (pTwist Amp[r] *HOF1-P3A* fragment (1264 bp before stop codon (position1254 in CDS) to 118 bp in 3'UTR)

AA substitutions, 337:S->A; 341:T->A;349:S->A 350:T->A; 364:T->A; 366:S->A; 369:S->A; 370:S->A; 381:S->A; 423:S->A; 424:S->A; 437:S->A; 450:T->A; 498:T->A; 516:T->A; 517:S->A; 518:T->A; 519:S->A; 520:T->A

Codon substitutions, 1009:TCT->GCT; 1021:ACC->GCT; 1045:TCC->GCT; 1048:ACA->GCT; 1090:ACA->GCT; 1096:TCT->GCT; 1105:TCT->GCT; 1108:TCA->GCT; 1141:TCC->GCT; 1267:AGC->GCT; 1270:TCA->GCT; 1309:AGC->GCT; 1348:ACA->GCT; 1491:ACG->GCT; 1546:ACC->GCT; 1549:AGT->GCA; 1552:ACA->GCT; 1555:AGC->GCT; 1558:ACA->GCT.

GTTAACAAATCATCTGAACTCATTCACGGCAGCGGATGAAATATCGACGTTTTCAAAAGAAAATGGAACTGGTAGACTGAAACATAAGACTT
CCAAAGGTGACATGAATTCTAGCGCCAACTGGGCTCAAATGAGCAGTATCTCCACGACAAGTAAAAAAACCGAATCGTATATGGACAATATA
AGGAAACTATCTTCTCAGCTGAAGGAAACCGAAAATAAAAGGAAACTAGCTTCAATAGACAAATATGAGAAACCATTGCCAGCTCCAGAAGT
CGCTATGGCTACACAATTTAGAAATGCTGCTCCCGTCATACGTAATGAGACAAAAGTGGTTGCTAACCCAGCTTTAGCTTTGAGGGCTGCTC
CCGTCCAGTTACAGAGCAATGTGGATGACGCTGTGTTAAGGCAGAAACCTGATAAACCAAGGCCGATTGTTGGGGAAGAACAACTTAAACC
TGATGAAGATTCGAAAAATCCTGATGAAAAAGGTCTAATGGTGCATAAAAGAAATCAATCTCTCGCTGCTCCATCAGAATCAAGTTCTTCTAA
TCCAACGGATTTTGCTCACATCAAAAAGAGACAAAGTATGGAATCTATGACTGCTTCCGTAAGTTCAATGGCCAATAGTATAGACGACTCAC
AGAGATTTGCCAAATCATGGAACTCGTCAAATAGGAAAAGAAAATCTATGAGCCATTTGCAAGTACCCTCATCCGCTTCTTCGAGGTCGGAT
GATGGGGGAAGAGCTCCTAACTCTGCGCACAACCTCAATGAAGATGATTATAACACGAGGAGAGATGCTGCAGCTGCTGCTATTTTGTTCA
AACCTCCTGTGGCAGTAAGAGGAACGTCTAGGGGGCACACGCATAGACAATCCATGATAATGCAAGATTCAAGTAATCCAATTGAAGATGC
CTTGTACGAAATGGAAAGAATCCAAAGTAGTTCTAAACCAGGTACAAAAACAGGAAACATCATGGATGAGAGAGGTGTGGTCAGGGATAGA
GGTATTACTGTTACCTTGCCTATTGTCACCAGTGAAGGTTTTCCAGTCATTGAATATGCCAAGGCCATGTATCCATTGATTGGAAATGAGGC
ACCTGGATTAGCGAATTTCCATAAGGGCGACTATCTGCTAATTACTGAGATTGTTAATAAAGATTGGTATAAAGGTGAAGTTTATGATAATGA
CCGTATAGATAGAAATCATAGGATAGGATTAATTCCCTATAATTTCATTCAGCTACTGCATCAAGGTCTT**TGA**TCTCGATGTATATCAATTTTA
CTAGTTTTCTGATAAAAGAAAAAAAGTATAATGTCAGATTTTTGTTAAGTAGAAATAGAAAGATATACGCTGCCGTGGGAGAAGGACACCTCT
CCGG

pSP1700 (pTwist Amp^r *HOF1-P3E* fragment (1264 bp before stop codon (position1254 in CDS) to 121 bp in 3'UTR)

AA substitutions, 337:S->E; 341:T->E; 349:S->E; 350:T->E; 364:T->E; 366:S->E; 369:S->E; 370:S->E; 381:S->E; 423:S->E; 424:S->E; 437:S->E; 450:T->E; 498:T->E; 516:T->E; 517:S->E; 518:T->E; 519:S->E; 520:T->E

Codon substitutions, 1009:TCT->GAA; 1021:ACC->GAA; 1045:TCC->GAA; 1048:ACA->GAA; 1090:ACA->GAA; 1096:TCT->GAA; 1105:TCT->GAA; 1108:TCA->GAA; 1141:TCC->GAA; 1267:AGC->GAA; 1270:TCA->GAA; 1309:AGC->GAA; 1348:ACA->GAA; 1491:ACG->GAA; 1546:ACC->GAG; 1549:AGT->GAG; 1552:ACA->GAA; 1555:AGC->GAA; 1558:ACA->GAA.

GTTAACAAATCATCTGAACTCATTCACGGCAGCGGATGAAATATCGACGTTTTCAAAAGAAAATGGAACTGGTAGACTGAAACATAAGACTT
CCAAAGGTGACATGAATTCTAGCGCCAACTGGGCTCAAATGAGCAGTATCTCCACGACAAGTAAAAAAACCGAATCGTATATGGACAATATA
AGGAAACTATCTTCTCAGCTGAAGGAAACCGAAAATAAAAGGAAACTAGCTTCAATAGACAAATATGAGAAACCATTGCCAGAACCAGAAGT
CGAAATGGCTACACAATTTAGAAATGAAGAACCCGTCATACGTAATGAGACAAAAGTGGTTGCTAACCCAGAATTAGAATTGAGGGAAGAAC
CCGTCCAGTTACAGAGCAATGTGGATGACGAAGTGTTAAGGCAGAAACCTGATAAACCAAGGCCGATTGTTGGGGAAGAACAACTTAAACC
TGATGAAGATTCGAAAAATCCTGATGAAAAAGGTCTAATGGTGCATAAAAGAAATCAATCTCTCGAAGAACCATCAGAATCAAGTTCTTCTAA

TCCAACGGATTTTGAACACATCAAAAAGAGACAAAGTATGGAATCTATGACTGAATCCGTAAGTTCAATGGCCAATAGTATAGACGACTCAC
AGAGATTTGCCAAATCATGGAACTCGTCAAATAGGAAAAGAAAATCTATGAGCCATTTGCAAGTACCCTCATCCGCTTCTTCGAGGTCGGAT
GATGGGGGAAGAGAACCTAACTCTGCGCACAACCTCAATGAAGATGATTATAACACGAGGAGAGATGAGGAGGAAGAAGAAATTTTGTTCA
AACCTCCTGTGGCAGTAAGAGGAACGTCTAGGGGGCACACGCATAGACAATCCATGATAATGCAAGATTCAAGTAATCCAATTGAAGATGC
CTTGTACGAAATGGAAAGAATCCAAAGTAGTTCTAAACCAGGTACAAAAACAGGAAACATCATGGATGAGAGAGGTGTGGTCAGGGATAGA
GGTATTACTGTTACCTTGCCTATTGTCACCAGTGAAGGTTTTCCAGTCATTGAATATGCCAAGGCCATGTATCCATTGATTGGAAATGAGGC
ACCTGGATTAGCGAATTTCCATAAGGGCGACTATCTGCTAATTACTGAGATTGTTAATAAAGATTGGTATAAAGGTGAAGTTTATGATAATGA
CCGTATAGATAGAAATCATAGGATAGGATTAATTCCCTATAATTTCATTCAGCTACTGCATCAAGGTCTT**TGA**TCTCGATGTATATCAATTTTA
CTAGTTTTCTGATAAAAGAAAAAAAGTATAATGTCAGATTTTTGTTAAGTAGAAATAGAAAGATATACGCTGCCGTGGGAGAAGGACACCTCT
CCGG

pSP1721 (pTwist Amp^r *HOF1-P4A* fragment (1264 bp before stop codon (position1254 in CDS) to 121 bp in 3'UTR)

AA substitutions, 313:S->A; 314:S->A; 337:S->A; 341:T->A; 350:T->A; 366:S->A; 421:S->A; 423:S->A; 424:S->A; 563:S->A.

Codon substitutions, 937:TCT->GCT; 940:TCT->GCT; 1009:TCT->GCT; 1021:ACC->GCT; 1048:ACA->GCT; 1096:TCT->GCT; 1261:TCT->GCT; 1267:AGC->GCT; 1270:TCA->GCT; 1687:AGT->GCT.

GTTAACAAATCATCTGAACTCATTCACGGCAGCGGATGAAATATCGACGTTTTCAAAAGAAAATGGAACTGGTAGACTGAAACATAAGACTT
CCAAAGGTGACATGAATTCTAGCGCCAACTGGGCTCAAATGAGCAGTATCTCCACGACAAGTAAAAAAACCGAATCGTATATGGACAATATA
AGGAAACTAGCTGCTCAGCTGAAGGAAACCGAAAATAAAAGGAAACTAGCTTCAATAGACAAATATGAGAAACCATTGCCAGCTCCAGAAGT
CGCTATGGCTACACAATTTAGAAATTCCGCTCCCGTCATACGTAATGAGACAAAAGTGGTTGCTAACCCAACATTAGCTTTGAGGTCTTCAC
CCGTCCAGTTACAGAGCAATGTGGATGACTCCGTGTTAAGGCAGAAACCTGATAAACCAAGGCCGATTGTTGGGGAAGAACAACTTAAACC
TGATGAAGATTCGAAAAATCCTGATGAAAAAGGTCTAATGGTGCATAAAAGAAATCAAGCTCTCGCTGCTCCATCAGAATCAAGTTCTTCTAA
TCCAACGGATTTTAGCCACATCAAAAAGAGACAAAGTATGGAATCTATGACTACATCCGTAAGTTCAATGGCCAATAGTATAGACGACTCAC
AGAGATTTGCCAAATCATGGAACTCGTCAAATAGGAAAAGAAAATCTATGAGCCATTTGCAAGTACCCTCATCCGCTTCTTCGAGGTCGGAT
GATGGGGGAAGAACGCCTAACTCTGCGCACAACCTCAATGAAGATGATTATAACACGAGGAGAGATACCAGTACAAGCACAATTTTGTTCA
AACCTCCTGTGGCAGTAAGAGGAACGTCTAGGGGGCACACGCATAGACAATCCATGATAATGCAAGATTCAAGTAATCCAATTGAAGATGC
CTTGTACGAAATGGAAAGAATCCAAGCTAGTTCTAAACCAGGTACAAAAACAGGAAACATCATGGATGAGAGAGGTGTGGTCAGGGATAGA
GGTATTACTGTTACCTTGCCTATTGTCACCAGTGAAGGTTTTCCAGTCATTGAATATGCCAAGGCCATGTATCCATTGATTGGAAATGAGGC
ACCTGGATTAGCGAATTTCCATAAGGGCGACTATCTGCTAATTACTGAGATTGTTAATAAAGATTGGTATAAAGGTGAAGTTTATGATAATGA
CCGTATAGATAGAAATCATAGGATAGGATTAATTCCCTATAATTTCATTCAGCTACTGCATCAAGGTCTTTGATCTCGATGTATATCAATTTTA
CTAGTTTTCTGATAAAAGAAAAAAAGTATAATGTCAGATTTTTGTTAAGTAGAAATAGAAAGATATACGCTGCCGTGGGAGAAGGACACCTCT
CCGG

pSP1722 (pTwist Amp^r *HOF1-P4E* fragment (1264 bp before stop codon (position1254 in CDS) to 121 bp in 3'UTR)

AA substitutions, 313:S->E; 314:S->E; 337:S->E; 341:T->E; 350:T->E; 366:S->E; 421:S->E; 423:S->E; 424:S->E; 563:S->E.

Codon substitutions, 937:TCT->GAA; 940:TCT->GAA; 1009:TCT->GAA; 1021:ACC->GAA; 1048:ACA->GAA; 1096:TCT->GAA; 1261:TCT->GAA; 1267:AGC->GAG; 1270:TCA->GAA; 1687:AGT->GAA.

GTTAACAAATCATCTGAACTCATTCACGGCAGCGGATGAAATATCGACGTTTTCAAAAGAAAATGGAACTGGTAGACTGAAACATAAGACTT
CCAAAGGTGACATGAATTCTAGCGCCAACTGGGCTCAAATGAGCAGTATCTCCACGACAAGTAAAAAAACCGAATCGTATATGGACAATATA
AGGAAACTAGAAGAACAGCTGAAGGAAACCGAAAATAAAAGGAAACTAGCTTCAATAGACAAATATGAGAAACCATTGCCAGAACCAGAAGT
CGAAATGGCTACACAATTTAGAAATTCCGAACCCGTCATACGTAATGAGACAAAAGTGGTTGCTAACCCAACATTAGAATTGAGGTCTTCAC
CCGTCCAGTTACAGAGCAATGTGGATGACTCCGTGTTAAGGCAGAAACCTGATAAACCAAGGCCGATTGTTGGGGAAGAACAACTTAAACC
TGATGAAGATTCGAAAAATCCTGATGAAAAAGGTCTAATGGTGCATAAAAGAAATCAAGAACTCGAGGAACCATCAGAATCAAGTTCTTCTAA
TCCAACGGATTTTAGCCACATCAAAAAGAGACAAAGTATGGAATCTATGACTACATCCGTAAGTTCAATGGCCAATAGTATAGACGACTCAC
AGAGATTTGCCAAATCATGGAACTCGTCAAATAGGAAAAGAAAATCTATGAGCCATTTGCAAGTACCCTCATCCGCTTCTTCGAGGTCGGAT
GATGGGGGAAGAACGCCTAACTCTGCGCACAACCTCAATGAAGATGATTATAACACGAGGAGAGATACCAGTACAAGCACAATTTTGTTCA
AACCTCCTGTGGCAGTAAGAGGAACGTCTAGGGGGCACACGCATAGACAATCCATGATAATGCAAGATTCAAGTAATCCAATTGAAGATGC
CTTGTACGAAATGGAAAGAATCCAAGAAAGTTCTAAACCAGGTACAAAAACAGGAAACATCATGGATGAGAGAGGTGTGGTCAGGGATAGA
GGTATTACTGTTACCTTGCCTATTGTCACCAGTGAAGGTTTTCCAGTCATTGAATATGCCAAGGCCATGTATCCATTGATTGGAAATGAGGC
ACCTGGATTAGCGAATTTCCATAAGGGCGACTATCTGCTAATTACTGAGATTGTTAATAAAGATTGGTATAAAGGTGAAGTTTATGATAATGA
CCGTATAGATAGAAATCATAGGATAGGATTAATTCCCTATAATTTCATTCAGCTACTGCATCAAGGTCTTTGATCTCGATGTATATCAATTTTA
CTAGTTTTCTGATAAAAGAAAAAAAGTATAATGTCAGATTTTTGTTAAGTAGAAATAGAAAGATATACGCTGCCGTGGGAGAAGGACACCTCT
CCGG

pSP1723 (pTwist Amp^r *HOF1-P5A* fragment (1065 bp before stop codon (position1453 of CDS) to 121 bp in 3'UTR)

AA substitutions, 369:S->A; 370:S->A; 478:S->A.

Codon substitutions, 1105:TCT->GCC; 1108:TCA->GCT; 1432:TCT->GCT.

CAGCTGAAGGAAACCGAAAATAAAAGGAAACTAGCTTCAATAGACAAATATGAGAAACCATTGCCATCTCCAGAAGTCACCATGGCTACACA
ATTTAGAAATTCCACACCCGTCATACGTAATGAGACAAAAGTGGTTGCTAACCCAACATTATCTTTGAGGGCCGCTCCCGTCCAGTTACAGA
GCAATGTGGATGACTCCGTGTTAAGGCAGAAACCTGATAAACCAAGGCCGATTGTTGGGGAAGAACAACTTAAACCTGATGAAGATTCGAA
AAATCCTGATGAAAAAGGTCTAATGGTGCATAAAAGAAATCAATCTCTCAGCTCACCATCAGAATCAAGTTCTTCTAATCCAACGGATTTTAG

CCACATCAAAAAGAGACAAAGTATGGAATCTATGACTACATCCGTAAGTTCAATGGCCAATAGTATAGACGACTCACAGAGATTTGCCAAAT
CATGGAACTCGTCAAATAGGAAAAGAAAAGCTATGAGCCATTTGCAAGTACCCTCATCCGCTTCTTCGAGGTCGGATGATGGGGGAAGAAC
GCCTAACTCTGCGCACAACCTCAATGAAGATGATTATAACACGAGGAGAGATACCAGTACAAGCACAATTTTGTTCAAACCTCCTGTGGCAG
TAAGAGGAACGTCTAGGGGGCACACGCATAGACAATCCATGATAATGCAAGATTCAAGTAATCCAATTGAAGATGCCTTGTACGAAATGGAA
AGAATCCAAAGTAGTTCTAAACCAGGTACAAAAACAGGAAACATCATGGATGAGAGAGGTGTGGTCAGGGATAGAGGTATTACTGTTACCTT
GCCTATTGTCACCAGTGAAGGTTTTCCAGTCATTGAATATGCCAAGGCCATGTATCCATTGATTGGAAATGAGGCACCTGGATTAGCGAATT
TCCATAAGGGCGACTATCTGCTAATTACTGAGATTGTTAATAAAGATTGGTATAAAGGTGAAGTTTATGATAATGACCGTATAGATAGAAATC
ATAGGATAGGATTAATTCCCTATAATTTCATTCAGCTACTGCATCAAGGTCTTTGATCTCGATGTATATCAATTTTACTAGTTTTCTGATAAAA
GAAAAAAAGTATAATGTCAGATTTTTGTTAAGTAGAAATAGAAAGATATACGCTGCCGTGGGAGAAGGACACCTCTCCGG

pSP1724 (pTwist Amp^r *HOF1-P5E* fragment (1065 bp before stop codon (position 1453 of CDS) to 121 bp in 3'UTR)

AA substitutions, 369:S->E; 370:S->E; 478:S->E.

Codon substitutions, 1105:TCT->GAA; 1108:TCA->GAG; 1432:TCT->GAA.

CAGCTGAAGGAAACCGAAAATAAAAGGAAACTAGCTTCAATAGACAAATATGAGAAACCATTGCCATCTCCAGAAGTCACCATGGCTACACA
ATTTAGAAATTCCACACCCGTCATACGTAATGAGACAAAAGTGGTTGCTAACCCAACATTATCTTTGAGGGAAGAGCCCGTCCAGTTACAGA
GCAATGTGGATGACTCCGTGTTAAGGCAGAAACCTGATAAACCAAGGCCGATTGTTGGGGAAGAACAACTTAAACCTGATGAAGATTCGAA
AAATCCTGATGAAAAAGGTCTAATGGTGCATAAAAGAAATCAATCTCTCAGCTCACCATCAGAATCAAGTTCTTCTAATCCAACGGATTTTAG
CCACATCAAAAAGAGACAAAGTATGGAATCTATGACTACATCCGTAAGTTCAATGGCCAATAGTATAGACGACTCACAGAGATTTGCCAAAT
CATGGAACTCGTCAAATAGGAAAAGAAAAGAAATGAGCCATTTGCAAGTACCCTCATCCGCTTCTTCGAGGTCGGATGATGGGGGAAGAAC
GCCTAACTCTGCGCACAACCTCAATGAAGATGATTATAACACGAGGAGAGATACCAGTACAAGCACAATTTTGTTCAAACCTCCTGTGGCAG
TAAGAGGAACGTCTAGGGGGCACACGCATAGACAATCCATGATAATGCAAGATTCAAGTAATCCAATTGAAGATGCCTTGTACGAAATGGAA
AGAATCCAAAGTAGTTCTAAACCAGGTACAAAAACAGGAAACATCATGGATGAGAGAGGTGTGGTCAGGGATAGAGGTATTACTGTTACCTT
GCCTATTGTCACCAGTGAAGGTTTTCCAGTCATTGAATATGCCAAGGCCATGTATCCATTGATTGGAAATGAGGCACCTGGATTAGCGAATT
TCCATAAGGGCGACTATCTGCTAATTACTGAGATTGTTAATAAAGATTGGTATAAAGGTGAAGTTTATGATAATGACCGTATAGATAGAAATC
ATAGGATAGGATTAATTCCCTATAATTTCATTCAGCTACTGCATCAAGGTCTTTGATCTCGATGTATATCAATTTTACTAGTTTTCTGATAAAA
GAAAAAAAGTATAATGTCAGATTTTTGTTAAGTAGAAATAGAAAGATATACGCTGCCGTGGGAGAAGGACACCTCTCCGG

pSP1937 (pTwist Amp^r *HOF1(F-BAR1)* fragment (-421 bp in 5'UTR to 316 bp in CDS)

AA substitutions: 39: K->E; 41: R->E; 58: K->E

Codon substitutions: 115: AAG->GAG; 121: CGT->GAA; 172: AAA->GAA

ATAAACATTGGTGATATACTGCAAGCAACAAGGAGTTCTCCCCATCGAGTGGCAGTAGTTCTTCCCCCTCTCGAGGGTGCCCCTTTCGGTA
GTTTGCGATTTTCTTCCAGAAGAGCACCGATTGCCCCATCCGGAAAGTACTATTTCCCTTTTGGGTAACAGCGGACCGAAGCGGAAGCGTT
CGAGTAGCCGAGGTATATGATTTCCTCTTTGGGCAAGTTGTAAACAATAAATGGTAAAAAGTTATGAAATATATTCAATGGTAAAGGTCCATT
TGATAAAGTACACATTCACTGTATGAGGGAAAGAGGAAAGCCAGCTAGAGCATTTTTTTGGAGCAGGCGAGCAAACAATATAAAATACAAAG
GCCTCGTGTGAGACTTGGAAAGTGTACTACTAATATTCAGAAAAAGGTGAAAGAATGAGCTACAGTTATGAAGCTTGTTTTTGGGACCCAAA
CGACAATGGTGTGAACATCCTTCTGGGTCACATTTCTCAGGGAATAAGATCTTGCGACTCAATGATACTTTTCTTTGAGCAGGAAAGTGAGC
TTGAGAAGGACTATGCCAGGCGGCTTGGAGCCATCACGGGAGAACTAGACAAAGACATTGGAACAAACATGGATTATGGAAAGTTGAATGA
AACATTTAATGTGGTGCTCAGTGTTGAGAAAGCTCGAGCACAATCGCATTCCAAGCAAAGTGAGATTCTTTTCAGACAGATTTACACGGATA
CTA

pSP1938 (pTwist Amp^r *HOF1(F-BAR2)* fragment (-421 bp in 5'UTR to 316 bp in CDS)

AA substitutions: 46: K->E; 50: R->E; 51: R->E

Codon substitutions: 136: AAG->GAG; 148: AGG->GAA; 151: CGG->GAG

ATAAACATTGGTGATATACTGCAAGCAACAAGGAGTTCTCCCCATCGAGTGGCAGTAGTTCTTCCCCCTCTCGAGGGTGCCCCTTTCGGTA
GTTTGCGATTTTCTTCCAGAAGAGCACCGATTGCCCCATCCGGAAAGTACTATTTCCCTTTTGGGTAACAGCGGACCGAAGCGGAAGCGTT
CGAGTAGCCGAGGTATATGATTTCCTCTTTGGGCAAGTTGTAAACAATAAATGGTAAAAAGTTATGAAATATATTCAATGGTAAAGGTCCATT
TGATAAAGTACACATTCACTGTATGAGGGAAAGAGGAAAGCCAGCTAGAGCATTTTTTTGGAGCAGGCGAGCAAACAATATAAAATACAAAG
GCCTCGTGTGAGACTTGGAAAGTGTACTACTAATATTCAGAAAAAGGTGAAAGAATGAGCTACAGTTATGAAGCTTGTTTTTGGGACCCAAA
CGACAATGGTGTGAACATCCTTCTGGGTCACATTTCTCAGGGAATAAGATCTTGCGACTCAATGATACTTTTCTTTAAGCAGCGTAGTGAGC
TTGAGGAGGACTATGCCGAAGAGCTTGGAGCCATCACGGGAAAACTAGACAAAGACATTGGAACAAACATGGATTATGGAAAGTTGAATGA
AACATTTAATGTGGTGCTCAGTGTTGAGAAAGCTCGAGCACAATCGCATTCCAAGCAAAGTGAGATTCTTTTCAGACAGATTTACACGGATA
CTA

pSP1939 (pTwist Amp^r *HOF1(F-BAR3)* fragment (-421 bp in 5'UTR to 462 bp in CDS)

AA substitutions: 99: R->E; 106: K->E

Codon substitutions: 295: AGA->GAA; 316: AAG->GAG

ATAAACATTGGTGATATACTGCAAGCAACAAGGAGTTCTCCCCATCGAGTGGCAGTAGTTCTTCCCCCTCTCGAGGGTGCCCCTTTCGGTA
GTTTGCGATTTTCTTCCAGAAGAGCACCGATTGCCCCATCCGGAAAGTACTATTTCCCTTTTGGGTAACAGCGGACCGAAGCGGAAGCGTT
CGAGTAGCCGAGGTATATGATTTCCTCTTTGGGCAAGTTGTAAACAATAAATGGTAAAAAGTTATGAAATATATTCAATGGTAAAGGTCCATT
TGATAAAGTACACATTCACTGTATGAGGGAAAGAGGAAAGCCAGCTAGAGCATTTTTTTGGAGCAGGCGAGCAAACAATATAAAATACAAAG

GCCTCGTGTGAGACTTGGAAAGTGTACTACTAATATTCAGAAAAAGGTGAAAGAATGAGCTACAGTTATGAAGCTTGTTTTTGGGACCCAAA
CGACAATGGTGTGAACATCCTTCTGGGTCACATTTCTCAGGGAATAAGATCTTGCGACTCAATGATACTTTTCTTTAAGCAGCGTAGTGAGC
TTGAGAAGGACTATGCCAGGCGGCTTGGAGCCATCACGGGAAAACTAGACAAAGACATTGGAACAAACATGGATTATGGAAAGTTGAATGA
AACATTTAATGTGGTGCTCAGTGTTGAGAAAGCTCGAGCACAATCGCATTCCAAGCAAAGTGAGATTCTTTTCGAACAGATTTACACGGATA
CTGAGGCATTTGCCGCTAACTTGCAAGCAAGATATACTACATTGAGTGGAAAGATTGAAAGGTTGCGGATGGACAAGTTCAACAAGAAAAA
GGGGTGTGAGGTGTTGCAAAAGAAATTACAGGATGCCCAGATTAGATTCAGAGACTTG
